# Significant out-of-sample classification from methylation profile scoring for amyotrophic lateral sclerosis

**DOI:** 10.1038/s41525-020-0118-3

**Published:** 2020-02-27

**Authors:** Marta F. Nabais, Tian Lin, Beben Benyamin, Kelly L. Williams, Fleur C. Garton, Anna A. E. Vinkhuyzen, Futao Zhang, Costanza L. Vallerga, Restuadi Restuadi, Anna Freydenzon, Ramona A. J. Zwamborn, Paul J. Hop, Matthew R. Robinson, Jacob Gratten, Peter M. Visscher, Eilis Hannon, Jonathan Mill, Matthew A. Brown, Nigel G. Laing, Karen A. Mather, Perminder S. Sachdev, Shyuan T. Ngo, Frederik J. Steyn, Leanne Wallace, Anjali K. Henders, Merrilee Needham, Jan H. Veldink, Susan Mathers, Garth Nicholson, Dominic B. Rowe, Robert D. Henderson, Pamela A. McCombe, Roger Pamphlett, Jian Yang, Ian P. Blair, Allan F. McRae, Naomi R. Wray

**Affiliations:** 1 Institute for Molecular Bioscience, The University of Queensland, Brisbane, QLD 4072 Australia; 2 University of Exeter Medical School, Royal Devon & Exeter Hospital, Exeter, Devon EX2 5DW UK; 3 Australian Centre for Precision Health, University of South Australia Cancer Research Institute, School of Health Sciences, University of South Australia, Adelaide, SA 5001 Australia; 4 Centre for Motor Neuron Disease Research, Macquarie University, Sydney, NSW 2109 Australia; 5 Brain Center Rudolf Magnus, University Medical Center Utrecht, Utrecht, 3584 CG Netherlands; 6 Mater Research Institute, The University of Queensland, Brisbane, QLD 4101 Australia; 7 Queensland Brain Institute, The University of Queensland, Brisbane, QLD 4072 Australia; 8 Institute of Psychiatry, Psychology & Neuroscience, King’s College London, London, SE5 8AF UK; 9 Australian Translational Genomics Centre, Queensland University of Technology, Brisbane, QLD 4102 Australia; 10 The Centre for Medical Research, Faculty of Health and Medical Sciences, The University of Western Australia, Nedlands, WA 6009 Australia; 11 Harry Perkins Institute of Medical Research, QEII Medical Centre, Nedlands, WA 6009 Australia; 12 Centre for Healthy Brain Ageing, School of Psychiatry, University of New South Wales, Sydney, NSW 2031 Australia; 13 Neuroscience Research Australia Institute, Randwick, NSW 2031 Australia; 14 Neuropsychiatric Institute, The Prince of Wales Hospital, University of New South Wales, Randwick, NSW 2031 Australia; 15 The Australian Institute for Bioengineering and Nanotechnology, The University of Queensland, Brisbane, QLD 4072 Australia; 16 Centre for Clinical Research, The University of Queensland, Brisbane, QLD 4019 Australia; 17 Fiona Stanley Hospital, Perth, WA 6150 Australia; 18 The University of Notre Dame Australia, Fremantle, WA 6160 Australia; 19 Institute for Immunology and Infectious Diseases, Murdoch University, Perth, WA 6150 Australia; 20 Calvary Health Care Bethlehem, Parkdale, VIC 3195 Australia; 21 ANZAC Research Institute, Concord Repatriation General Hospital, Sydney, NSW 2139 Australia; 22 Department of Neurology, Royal Brisbane and Women’s Hospital, Brisbane, QLD 4029 Australia; 23 Discipline of Pathology and Department of Neuropathology, Brain and Mind Centre, The University of Sydney, Sydney, NSW 2050 Australia

**Keywords:** Predictive markers, Amyotrophic lateral sclerosis

## Abstract

We conducted DNA methylation association analyses using Illumina 450K data from whole blood for an Australian amyotrophic lateral sclerosis (ALS) case–control cohort (782 cases and 613 controls). Analyses used mixed linear models as implemented in the OSCA software. We found a significantly higher proportion of neutrophils in cases compared to controls which replicated in an independent cohort from the Netherlands (1159 cases and 637 controls). The OSCA MOMENT linear mixed model has been shown in simulations to best account for confounders. When combined in a methylation profile score, the 25 most-associated probes identified by MOMENT significantly classified case–control status in the Netherlands sample (area under the curve, AUC = 0.65, CI_95%_ = [0.62–0.68], *p* = 8.3 × 10^−22^). The maximum AUC achieved was 0.69 (CI_95%_ = [0.66–0.71], *p* = 4.3 × 10^−34^) when cell-type proportion was included in the predictor.

## Introduction

Amyotrophic lateral sclerosis (ALS) is a severe neurodegenerative disease characterized by progressive muscle weakness and degeneration of upper and/or lower motor neurons in the central nervous system. Clinical heterogeneity in presentation of ALS^1^ may reflect a complex disease etiology, with considerable genetic contribution even among the >90% of cases that present without a strong family history of ALS.^1,2^ A lower bound for the genetic contribution is given by the proportion of variance associated with common single-nucleotide polymorphisms (SNPs) genome-wide, the SNP-based heritability estimated as 8.5% (CI_95%_ = [7.5–9.5]) in Europeans^3^ and 15% (CI_95%_ = [8–22]) in East Asians.^4^ Epidemiological studies have also identified environmental and occupational risk factors, such as metal and pesticide exposure^5^ and defense force occupation,^6,7^ but generally studies are underpowered.^8^ Hence, lifetime environmental exposures paired with genetic susceptibility likely contribute to an increased risk for ALS.

DNA methylation (DNAm), which in mammals is almost exclusively found in cytosine–guanine dinucleotides (CpG), is a widely studied epigenetic modification. Aberrant DNAm patterns can be consequence of environmental exposures, and/or cause or consequence of disease, and have been hypothesized to play a role in neurodegenerative diseases (including ALS).^9–12^ Indeed, a methylome-wide association study (MWAS) has suggested that neurodegenerative processes in ALS may be associated with DNAm alterations.^13^ In this study, the authors interrogated the methylation status of genome-wide CpG loci in postmortem spinal cord tissue. Despite the very small sample size (12 ALS subjects and 11 age and gender-matched neurologically normal controls), the authors reported 4261 significant differentially methylated positions (DMPs), annotated to 3574 genes. Functional enrichment analyses showed these genes to be highly enriched in biological functions related to immune and inflammation response. However, the presence of confounding factors, and failing to account for these confounders, has been widely recognized as a concern in MWAS,^14^ because they could lead to spurious association results. For example, Figueroa-Romero et al.^13^ did not account for the potential confounding of cell-type composition, as there is evidence that these explain much of the observed variability in DNAm.^15,16^ Nonetheless, identification of DMPs across the genome that may drive or be driven by ALS pathogenic processes remains of importance, especially for biomarker development.

Confounders in the context of DNAm studies need careful consideration. Some confounders such as sex and technical batch effects are usually recorded and can be modeled as covariates. However, other recognized lifestyle confounders may exist, but may not be recorded. Furthermore, there could be confounders that are present, but unknown. Some case–control DNAm differences, driven by cell-type composition, or changes due to environmental exposures, medications, or complications of the disease, may be considered confounders, or alternatively, may be considered of primary interest depending on the context of the scientific question. Some unmeasured confounders can be inferred from DNAm reference-based classification methods, for example the Horvath age predictor or the Houseman algorithm to predict cell-type proportions (CTP).^15,17^ However, predicted confounders have inherent classification error, and may only explain a proportion of the variation attributable to the directly measured covariates, which may result in inflated test statistics due to the uncaptured variation of confounders.^18^ More importantly, recent studies show that correction approaches (e.g., principal component analysis) widely used in standard MWAS and association studies of other molecular phenotypes may induce bias, which persists for large sample sizes and replicates out-of-sample.^19^

The OmicS-data-based Complex trait Analysis (OSCA) software^18^ implements mixed linear model (MLM) approaches: MLM-based omics association (MOA) and multi-component MLM-based omics association excluding the target (MOMENT). MOA and MOMENT analyses account for trait-associated probes that are highly correlated with other trait-associated probes across the genome, and assume effect sizes are drawn from a normal distribution. MOA assumes a single distribution of effects sizes, while MOMENT allows a different effect size distribution for the most-associated probes. Fewer trait-associated probes are identified in MOA and MOMENT analyses compared to linear regression, but simulations^18^ show that probes that are significantly associated from these analyses are more likely to be true positives. Briefly, the MOA method fits a random genome-wide DNAm factor per person with variance–covariance matrix (the omics relationship matrix or ORM) between individuals built from genome-wide DNAm sites (equivalent to a model of fitting all DNAm sites as random effects); this model is analogous to the MLM association method implemented in EMMAX^20^ and GCTA^21^ for SNP data. Extensive simulations showed high false-positive rate (FPR) in standard linear regression MWAS, while both MOA and MOMENT controlled for false discovery with little loss of power.^18^ However, in some scenarios, the more stringent MOMENT method is needed to control the FPR if a proportion of DNAm sites are much more correlated than the others, at the cost of slightly reduced power. The MOMENT method fits an MLM with two random-effect components for each probe tested (and hence two ORMs between subjects computed from two sets of DNAm sites), with the DNAm sites grouped by their associations with the trait (leaving out the DNAm sites in a window around the target probe being tested for association to avoid proximal contamination).^18^ Hence, the MOMENT method is more likely to identify DNAm differences that have a specific role in disease rather than reflect factors that impact multiple DNAm sites across the genome.

In this study, we applied both the MOA and MOMENT methods to a disease trait (ALS) and compared results with standard linear regression methods. We identified significant differences in predicted immune CTP and one significantly DMP between cases and controls. We used the estimated effect sizes of all DNAm sites, using best-linear unbiased prediction (BLUP) to calculate individual DNA methylation profile scores (MPS) in an independent ALS sample. The MPS classified ALS case–control status with area under the curve (AUC) of 0.61, CI_95%_ = [0.58–0.63], *p* = 5.6 × 10^−11^. The classification accuracy increased when the effects associated with predicted immune CTP were included (AUC = 0.69, CI_95%_ = [0.66–0.71], *p* = 4.3 × 10^−34^).

## Results

### Differences in predicted cell type proportions between ALS cases and controls

**Fig. 1:**
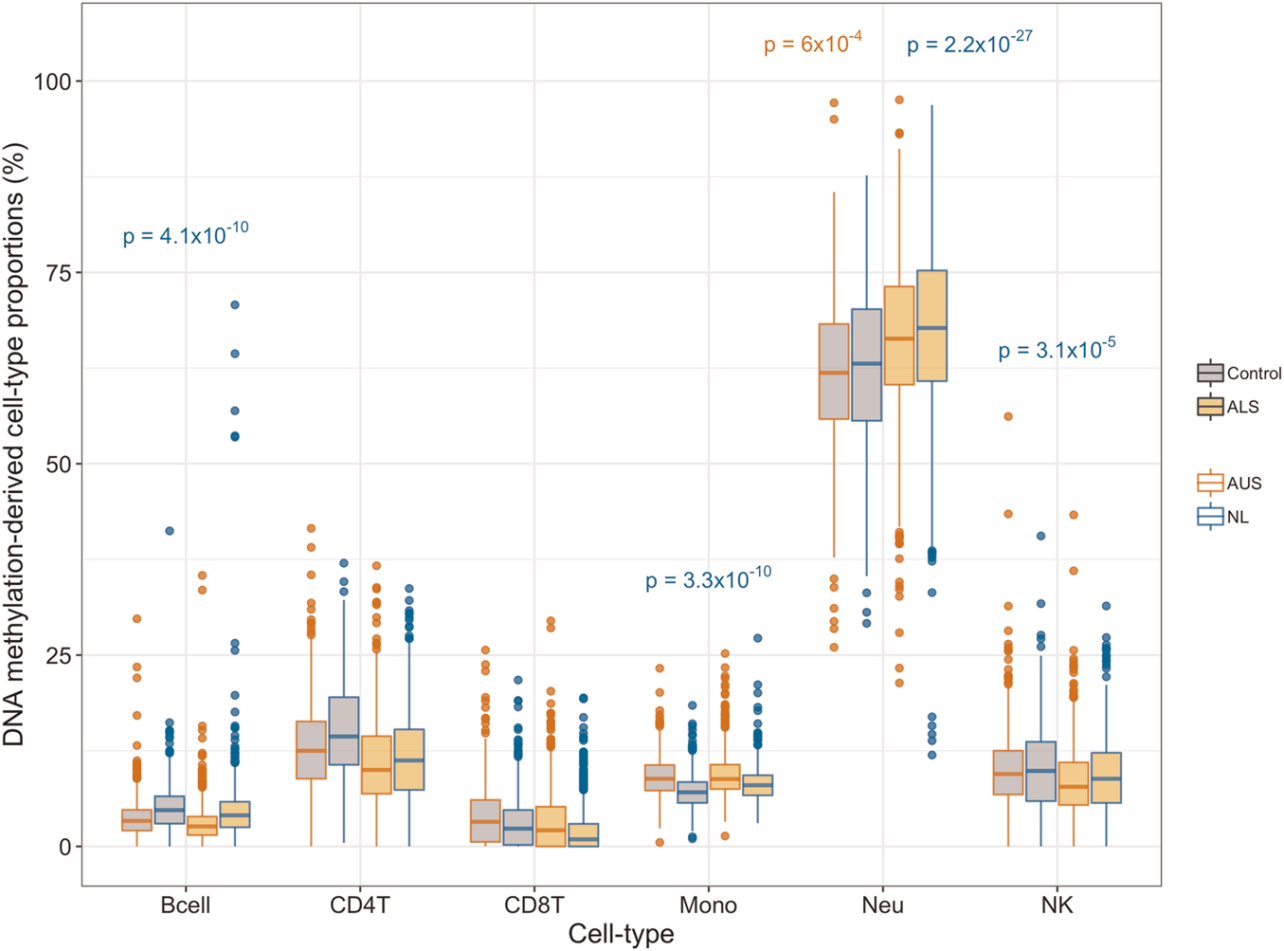
Predicted proportions of cell types estimated with the Houseman algorithm, which are based on methylation values of purified cell types from a whole-blood sample. Methylation-derived predicted cell proportions in % (*y*-axis) for different cell types (*x*-axis) in the Australian ALS case–control cohort (*N*_cases_ = 613 and *N*_controls_ = 782, red colored boxplots) and in the Netherlands ALS case–control cohort (*N*_cases_ = 1159 and *N*_controls_ = 637, blue colored boxplots). Gray—controls, orange—cases. *P* values are from stepwise logistic regression models (Methods) and indicate cell types significantly associated with case–control status, after Bonferroni correction. *P* values in red correspond to the AU ALS cohort and *p* values in blue correspond to the NL ALS cohort. The boxplot horizontal black line marks the median CTP value in that group. The lower and upper hinges correspond to the first and third quartiles (the 25th and 75th percentiles). The upper whisker extends from the hinge to the largest value no further than 1.5 IQR from the hinge (where IQR is the inter-quartile range, or distance between the first and third quartiles). The lower whisker extends from the hinge to the smallest value at most 1.5 IQR of the hinge. Data beyond the end of the whiskers are called “outlying” points and are plotted individually.

There is accumulating evidence for an active role of immune cells, and inflammation in general, on neurodegenerative disorders (both as cause and consequence of disease).^22,23^ Hence, our first analysis was to investigate differences in predicted CTP between cases and controls. We used the Houseman algorithm based on purified cell types from whole blood^15^ to estimate blood CTP for each individual based on the DNAm data. Using stepwise backward logistic regression models, increased neutrophil proportions were found to be significantly associated with ALS, after Bonferroni correction (OR = 1.02, CI_95%_ = [1.01–1.04], *p* = 6 × 10^−4^) (Fig. 1). In the Netherlands (NL) sample, the pattern of differences in CTP was similar to that of the Australian (AU) sample, and with its larger sample size neutrophils (OR = 1.11, CI_95%_ = [1.09–1.13], *p* = 2.2 × 10^−27^), monocytes (OR = 1.29, CI_95%_ = [1.22–1.36], *p* = 3.3 × 10^−20^), B lymphocytes (OR = 1.12, CI_95%_ = [1.08–1.16], *p* = 4.1 × 10^−10^), and natural killer cells (OR = 1.06, CI_95%_ = [1.03–1.1], *p* = 3.1 × 10^−5^) were all significantly associated with ALS (Fig. 1).

### DNA MWAS analysis

**Fig. 2:**
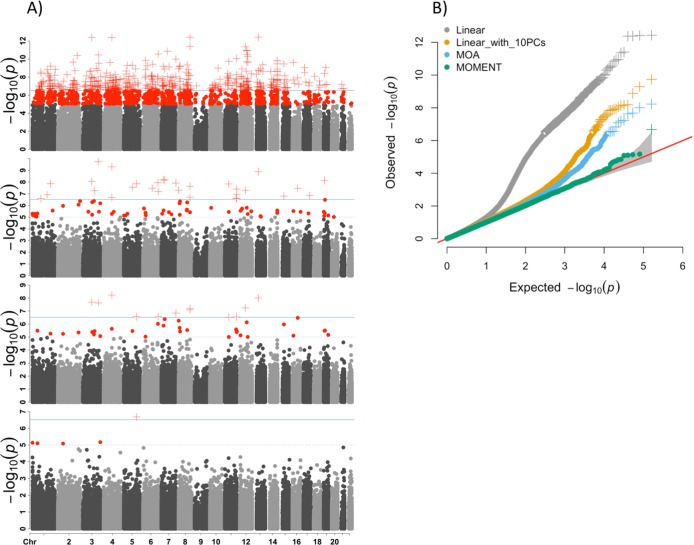
Manhattan and QQ plots of MWAS using linear or mixed linear regression models, for the Australian ALS samples (*N*_cases_ = 613 and *N*_controls_ = 782). **a** From top to bottom row: Manhattan plots using linear regression, linear regression with 10 principal components calculated from the ORM as fixed effects, MOA, and MOMENT. Red circles represent probes with *p* < 1 × 10^−5^; red crosses represent probes with *p* < 3.1 × 10^−7^ genome-wide significant threshold. Solid dark blue line mark *p* < 3.1 × 10^−7^ and dashed sky-blue line marks *p* < 1 × 10^−5^. **b** QQ-plots showing the expected and observed −log(*p*) in each model. We calculated the genomic inflation factor (*λ*) as the median of *χ*^2^ test statistics of all probes divided by its expected value under the null. *λ*_Linear_ = 1.19, *λ*_Linear_with_PCs_ = 1.1, *λ*_MOA_ = 1.01, *λ*_MOMENT_ = 1.02.

**Fig. 3:**
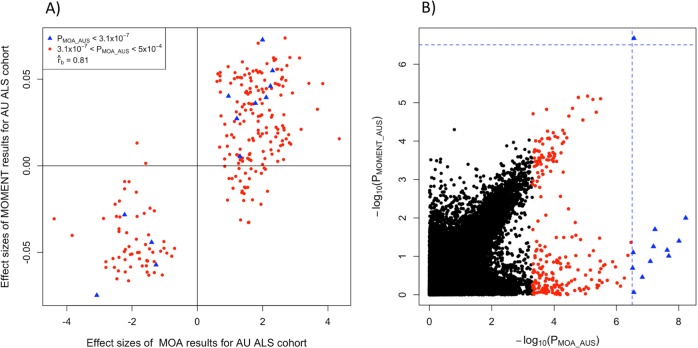
MWAS results from MOA and MOMENT show high correlation of effect sizes in the Australian ALS cohort. **a** −log10(*p*) of all probes in MOA (*x*-axis) and MOMENT (*y*-axis), for the AU ALS dataset. Dashed blue lines mark the genome-wide significance threshold (*p* = 3.1 × 10^−7^) of MOA and MOMENT. Red dots mark all probes with *p* < 5 × 10^−4^ from MOA (*m* = 241) as in **b.** Effect sizes of MOA (*x*-axis) and MOMENT (*y*-axis), for AU ALS dataset, of probes with *p* < 5 × 10^−4^ from MOA. Correlation of effect sizes: $\hat r_{\mathrm b} = 0.81$ = 0.81, s.e. = 0.03.

MWAS results show that under an MLM framework the number of significant DMPs is much reduced, compared to the standard linear regression models (Fig. 2a). At a Bonferroni corrected genome-wide significance threshold of *p* = 3.1 × 10^−7^, the number of DMPs passing significance are 476 in a linear model without any covariates; 30 in a linear model with the first 10 PCs, calculated from the ORM as fixed effects added to the model as covariates; 12 in MOA, and 1 in MOMENT (top to bottom row of Fig. 2a, respectively). The genomic inflation factor, *λ* (the median of *χ*^2^ test statistics of all DNAm sites divided by its expected value under the null), is better controlled using MOA and MOMENT compared to the linear regression models (Fig. 2b). The significance of almost all MOA-identified DNAm sites is reduced in MOMENT (Table 1, Fig. 3a), with the exception of cg04104695 annotated to gene *CXXC5*. However, MOA and MOMENT regression coefficients of DNAm sites with *p* from MOA < 5 × 10^−4^ (*m* = 241) are still highly correlated (Fig. 3b, $\hat r_{\mathrm b} = 0.81$, s.e. = 0.03). Interestingly, the correlation of effect sizes is much higher between standard linear regression models and MOA (Supplementary Fig. 1, $\hat r_{\mathrm b} = 1$, s.e. = 3 × 10^−3^) than with MOMENT ($\hat r_{\mathrm b} = - 0.2$, s.e. = 0.02), when considering probes that are significant from the linear regression model. From simulations, MOMENT analyses have been shown to be more powerful in controlling for potential confounders, with a caveat of a slight loss of power.^18^ In a previous MWAS analysis of lung function it could be shown that the probes associated with smoking, a confounder not included in the analysis, could explain the probes significantly associated in MOA, but not MOMENT analysis.^18^ Hence, the difference between the number of significant DMPs found by MOA or MOMENT could reflect a small difference in power for detection of true positives, or a higher number of false positives in MOA.

**Table 1:** DNA methylation sites significantly associated with ALS at *p* < 3.1 × 10^−7^ from MOA and MOMENT in the Australian cohort.

Chr	Probe	bp	Gene	Orientation	b_MOA	p_MOA	b_MOMENT	p_MOMENT
4	cg08422863	88379498	HERC6	R	−0.16	5.9 × 10^−9^	−0.07	0.01
13	cg05380910	38989909	STOML3	F	0.15	9.8 × 10^−9^	0.06	0.04
3	cg24866706	96619630	RPL18AP8;MTRNR2L12	R	0.15	2.1 × 10^−8^	0.04	0.09
3	cg27143246	169771795	MYNN;RP11−816J6.3	R	−0.15	2.4 × 10^−8^	−0.05	0.07
12	cg00178984	59697390	SLC16A7	R	0.15	5.8 × 10^−8^	0.06	0.02
8	cg26078251	124300968	RP11-383J24.2	F	0.15	6.5 × 10^−8^	0.05	0.06
8	cg20134271	124483581	RNF139	R	0.15	8.2 × 10^−8^	0.04	0.13
7	cg05303559	158719689	AC019084.7	F	0.14	1.5 × 10^−7^	0.03	0.35
5	cg04104695	139679164	CXXC5	R	−0.14	2.7 × 10^−7^	−0.14	2.1 × 10^−7^
6	cg12785183	166031972	**—**	F	0.14	2.7 × 10^−7^	0.01	0.9
11	cg21836562	128868867	KCNJ1	R	0.14	2.9 × 10^−7^	0.05	0.08
11	cg07613278	43311777	API5	F	−0.14	3 × 10^−7^	−0.03	0.2

*Chr* chromosome number, *Probe* probe identification number as provided by Illumina, *bp* base pair position in the genome, *Gene* closest genes the probe is annotated to, based on distance to transcription starting site, following the method described elsewhere,^44^ *Orientation* DNA strand orientation, *F* forward, *R* Reverse, *b_MOA* effects sizes (increase (positive sign) or decrease (negative sign) of methylation between cases and controls per standard deviation unit) of AU MOA, *p_MOA* ps of MOA models for AU, *b_MOMENT* effects sizes (interpreted as b_MOA) of AU MOMENT, *p_MOMENT* ps of AU MOMENT.

The NL cohort was available to us to seek replication of our DMP. Of the 101 top DNAm sites (suggestive *p* < 5 × 10^−4^) in the AU MOMENT analysis, 94 were available in the NL sample; however, none replicated at *p* < 0.05/94, i.e. 5.3 × 10^−4^ (Supplementary Fig. 2a). Post hoc power calculations (Supplementary Fig. 2c, d) show that the replication sample size necessary to detect a true association based on the estimated effect size of the most-associated probe (i.e., cg04104695) would be 3478 (assuming a balanced design, 80% power and replication significance threshold *p* = 5.3 × 10^−4^). Hence, the lack of replication of individual probes may reflect lack of power. Consistent with this conclusion, we found the effect sizes of MOMENT results for the AU and NL samples for these 94 probes were correlated (Supplementary Fig. 2b, $\hat r_{\mathrm b} = 0.33$, s.e. = 0.2).

### Proportion of variance associated with genome-wide DNAm sites

To provide quantitative description of the relationship between case–control status and DNAm covariates, we estimated the proportion of variation in case–control status captured by genome-wide DNAm ($\hat \rho ^2$) by OREML (see Methods) using probe values corrected for known potential confounders. This approach parallels estimation of SNP-based heritability,^24^ except that SNP-based heritability represents the variance attributable to genome-wide SNPs and so has an inference of association through causality. In contrast, with DNAm (or other molecular phenotypes) the $\hat \rho ^2$ estimate could reflect both causes and consequences of disease (including consequences of medication). Therefore, it is not appropriate to transform this estimate to the liability scale (as is done for SNP-based heritability estimates). The estimate must be interpreted as dependent on the proportion of cases in the sample (which influences the phenotypic variance on this scale), and hence we report both $\hat \rho ^2$ and phenotypic variance $\left( {\hat \sigma _P^2} \right)$ (since phenotypic variance is reduced by inclusion of covariates) to aid interpretation of results. In the baseline model with no covariates, $\hat \sigma _P^2 = 0.247$ and $\hat \rho ^2 = 15\%$ (Table 2), and $\hat \sigma _P^2$ simply binomial variance given the proportion of the sample that are cases. A model with confounder covariates (predicted age + sex + smoking score + batch effects) gave $\hat \rho ^2 = 17\%$ ($\hat \sigma _P^2 = 0.227$, 92% of baseline $\hat \sigma _P^2$). A model with predicted CTP as fixed effects gives a higher $\hat \rho ^2 = 24\%$ ($\hat \sigma _P^2 = 0.237$, 96% of baseline $\hat \sigma _P^2$). A model with both the confounder covariates and predicted CTP as covariates gave $\hat \rho ^2 = 31\%$ ($\hat \sigma _P^2 = 0.221$, 89% of baseline $\hat \sigma _P^2$).

**Table 2:** Proportion of phenotypic variance captured by all DNAm sites $(\hat \rho ^2)$ and phenotypic variance $(\hat \sigma _P^2)$ estimated from different OREML models.

OREML model (*y* = Cβ + Wu + e)	$\hat \rho ^2$ (s.e.)	$\hat \sigma _P^2$ (s.e.)
No covariates	15% (0.05)	0.247 (0.01)
With predicted age + predicted smoking score + sex + batch effects	17% (0.06)	0.227 (0.01)
With predicted cell proportions	24% (0.06)	0.237 (0.01)
With predicted age + predicted smoking score + sex + batch effects + predicted cell proportions	31% (0.08)	0.221 (0.01)

*s.e.* standard error. In the absence of covariates $\hat \sigma _P^2$ = *P*(1 − *P*), where *P* is the proportion of the sample that are cases.

### Out-of-sample classification using DNA methylation profiles scores (MPS)

**Fig. 4:**
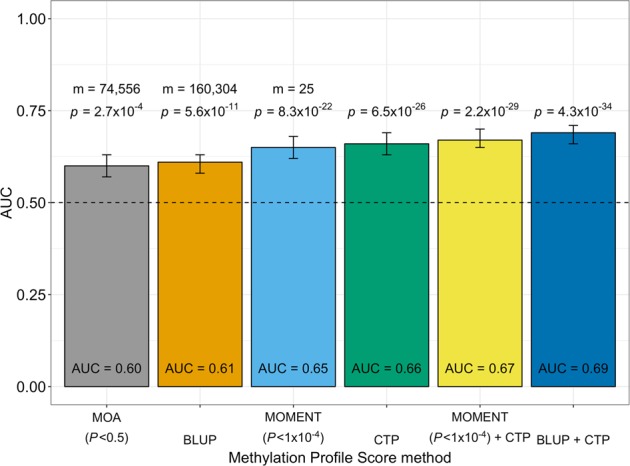
Maximum AUC given by the different methods used to calculate MPS, when classifying from the Australian (*N*_cases_ = 613, *N*_controls_ = 782) to the Netherlands (*N*_cases_ = 1159 and *N*_controls_ = 637) ALS cohort. Bars indicate 95% confidence intervals of AUC values for each method. MOA: CI_95%_ = [0.57–0.63]; BLUP: CI_95%_ = [0.58–0.63]; MOMENT: CI_95%_ = [0.62–0.68]; predicted cell proportions (CTP): CI_95%_ = [0.63–0.69]; MOMENT + CTP: CI_95%_ = [0.65–0.7]; and BLUP + CTP: CI_95%_ = [0.66–0.71]. Dashed line indicates AUC = 0.5, i.e., random classification. *P* values are from logistic regression models. *m* = number of DNAm probes used to calculate the MPS.

Out-of-sample classification provides independent evidence that differences in DNAm between cases and controls reflect differences associated with disease status rather than technical confounding effects, as the latter are less likely to be shared between independently collected and processed samples. It can also leverage DNAm differences between cases and controls that do not achieve statistical significance. MPS were calculated for each individual in the NL sample as the sum of DNAm probe values weighted by their effect sizes estimated in the AU sample (Supplementary Fig. 3). Figure 4 summarizes the maximum AUC given by each of the different methods used to calculate MPS.

The BLUP model provides jointly estimated effect sizes for each probe, but assumes probe effect sizes are drawn from a normal distribution. The AUC for AU as discovery cohort and NL as target was 0.61 (Supplementary Fig. 4, CI_95%_ = [0.58–0.63], *p* = 5.6 × 10^−11^, *p* from logistic regression model). We also assessed the effect on classification accuracy of MPS derived from estimated effect sizes (from OREML) attributed to each CTP. Indeed, CTP-derived MPS alone were a better classifier than BLUP scores (Supplementary Fig. 4, AUC = 0.66, CI_95%_ = [0.63–0.69], *p* = 6.5 × 10^−26^). Classification efficacy was further increased when both BLUP-derived DNAm effect sizes and CTP effect sizes were used together to calculate the MPS (Supplementary Fig. 4, AUC = 0.69, CI_95%_ = [0.66–0.71], *p* = 4.3 × 10^−34^).

Finally, we used out-of-sample classification to gain insight about the results from the linear regression vs MOA vs MOMENT analyses (Supplementary Table 1). MPS based on MOA estimated effect sizes from AU as discovery sample to NL as target sample gave maximum AUC = 0.60 (CI_95%_ = [0.57–0.63], *p* = 2.7 × 10^−4^), using all probes with *p* < 0.5 (*m* = 74,556). However, MPS based on MOMENT results gave higher AUC for all *p* value thresholds tested, despite fewer probes passing each significance threshold. The maximum MOMENT AUC was 0.65 (CI_95%_ = [0.62–0.68], *p* = 8.3 × 10^−22^) with probes with *p* < 1 × 10^−4^ (*m* = 25). A model that included MPS calculated with MOMENT-derived effect sizes and CTP effect sizes gave AUC = 0.67 (CI_95%_ = [0.65–0.70], *p* = 2.2 × 10^−29^). We note a negative correlation between MOA and MOMENT MPS, despite high correlation of effect sizes (Supplementary Fig. 5). Based on simulations, Zhang et al.^18^ found this property to be induced when non-causal probes were included in the calculation of MPS. For causal probes in simulations, the MOA and MOMENT-based classifiers were strongly positively correlated.

Based on the DNAm profile scoring analyses we concluded that the MOMENT analyses are more likely to have identified true positive associations, AUC = 0.65 from 25 probes (Supplementary Table 2). We investigated if the top DMPs from MOMENT (*p* < 1 × 10^−4^, *m* = 25) overlapped with brain mQTL regions^25^ (*p* < 1 × 10^−5^) and GWAS SNPs^26^ (*p* < 5 × 10^−8^). We found no evidence for overlap with GWAS signals (Supplementary Table 3), which likely reflects lack of power in both MWAS and GWAS, as has previously been observed for body-mass index (BMI).^27^

## Discussion

In this study we have conducted the largest MWAS to date for ALS and we use the new software OSCA that implements different MLM approaches specifically designed for omics data. We have presented results to help evaluation of the comparison of linear regression, MOA and MOMENT methods for a disease trait, recognizing that the potential for confounding of technical artefacts is much greater for a binary trait as compared to a quantitative trait.

Using the most stringent MOMENT model, we identified 1 DMP between ALS cases and controls, which is annotated to *CXXC5* on chromosome 5 (cg04104695, *p*_MOA_ = 2.7 × 10^−7^, *p*_MOMENT_ = 2.1 × 10^−7^, decreased blood DNA methylation associated with ALS). The association was not replicated in the independent NL study, but power analyses suggest this may reflect effect size and sample size, reminiscent of early underpowered GWAS for which increasing sample sizes have led to many new (highly replicated) discoveries in the past decade.^28^ Despite lack of replication of the most-associated probe, out-of-sample classification using effect sizes estimated from the 25 most-associated DNAm sites in the AU sample gave a classification AUC of 0.65 (*p* = 2.2 × 10^−29^) in the NL sample. We summarize literature evidence of a functional role for CXXC5 in neurodegeneration and functional annotation of 25 most-associated DNAm sites in a Supplementary Note. We found better out-of-sample classification when using DNAm effect sizes estimated from MOMENT compared to MOA or linear regression results, despite fewer probes detected at each association *p* value threshold. Our results support the recommendation of Zhang et al.^18^ to use the MOMENT over MOA model, since MOA results may include more false-positive associations.

The maximum out-of-sample classification was achieved using a score calculated from BLUP-estimated DNAm effect sizes together with effect size estimates for predicted CTP (AUC = 0.69, CI_95%_ = [0.66–0.71], *p* = 4.3 × 10^−34^). We are careful to use the term out-of-sample classification rather than prediction, because the blood samples available to us in both the AU and NL cohorts were not taken prior to diagnosis, hence the strength of classification may reflect consequences as well as causes of disease. The trans-national out-of-sample classification is a strength of our study, but we recognize that best-practice medication and clinical management are likely shared across countries. In these historically collected samples we have incomplete records of riluzole use, which was the only approved medication at the time of sample collection. Despite these caveats it is noteworthy that the AUC is much higher for DNAm classification (here AUC = 0.69 from a discovery sample of 782 cases and 613 controls) compared to SNP polygenic risk score prediction (AUC = 0.57 in our AU sample, unpublished data, based on a discovery sample of 20,806 cases and 59,804 controls^26^). Comparison of genetic and DNAm predictors for some complex traits also found much smaller discovery samples are needed for DNAm predictors.^27,29^ However, this will also depend on the underlying (epi)genetic architecture of the trait (for example, DNAm predictors explained little variation in height, but high variation in BMI compared to genetic predictors^27^).

Given the interest in developing early diagnostic biomarkers from blood it seems a worthwhile goal to collect blood samples from patients when they first present in neurology clinics, prior to diagnosis, treatment, and clinical management. Generating DNAm profiles from such samples would allow evaluation of DNAm as a biomarker diagnostic tool. Such data could also evaluate the sensitivity of the predictor in differentiating ALS from ALS mimics, i.e., people who present to neurology clinics with ALS-like symptoms, but who do not achieve ALS diagnosis (often after lengthy clinical evaluation). Differences in CTP between cases and controls is an important contributor to the out-of-sample classification, consistent with reports of aberrant activation of the peripheral immune system in ALS,^30,31^ and with reports of associations between elevated white blood cell count and increased ratio of circulating neutrophils to monocytes with more-rapid progression of ALS.^32,33^ Here, we predict CTP based on the cell-type-specific DNAm signatures,^15^ but direct measurement of blood cell types might generate more accurate classifiers. We estimate association effect sizes of >200,000 DNAm sites, and necessarily effect sizes are estimated with error. Larger sample sizes are needed to increase accuracy of estimation of DNAm effect sizes and to increase classification accuracy. Larger, carefully phenotyped sample collections could allow evaluations of classification scores based on a combination of genetic, DNAm, other “*omics*” and environmental risk factors.

Our study has several limitations. First, a perceived limitation of our study may be that blood is not a relevant tissue for understanding the biological mechanisms underlying ALS, due to the tissue-specificity of most DNAm patterns.^34–36^ However, blood is an easily accessible tissue which is relevant for generation of biomarkers associated with disease. Biological or environmental interpretation of DNAm associations can be made as downstream analyses after their discovery. Variation between people in DNAm controlled by SNP variation has been shown to have high correlation between brain and blood,^25,37^ which provides further support that blood may be an appropriate tissue for the goal of developing early diagnostic biomarkers. A second limitation is that the blood sample available to us from both the AU and NL cohorts had incomplete clinical records, both in relation to time since diagnosis and with respect to clinical management and treatment. Ongoing sample collections in both countries are now collecting such data more consistently.

In summary, we applied a new OSCA analysis pipeline to DNAm data measured in whole blood in an Australian ALS case–control cohort. The MOMENT method is expected to identify differences in DNAm associated with disease independent of confounding factors such as differences in cell-type proportion or smoking that generate widespread DNAm changes. An MPS calculated from the 25 most-associated probes identified by MOMENT, and an MPS based on cell-type differences both generate significant classification of ALS in an independent sample (Fig. 3), with the profile scores themselves being correlated only at 0.12 (Supplementary Fig. 5). Given our relatively small discovery sample, our significant classification results from an Australian discovery cohort into a Netherlands target cohort indicate that DNAm may be a useful predictive biomarker. We advocate for larger samples with blood collected prior to diagnosis and with deep clinical phenotyping to allow investigation of this proposal.

## Methods

### Datasets description

The Australian ALS cohort (AU) consisted of two cohorts, AU1 (440 cases, 418 controls) and AU2 (342 cases ALS, 195 controls) (Supplementary Table 4). For AU1, patients and controls were ascertained from the University of Sydney as part of the Australian MND DNA bank, which recruited participants from April 2000 to June 2011. Cases were white Australians older than 25 years recruited from around Australia via state-based MND associations with diagnoses verified by neurologists. All participants gave written consent and the study protocol was approved by the Sydney South West Area Health Service Human Research Ethics Committee (HREC). AU2 cases were recruited from clinics across Australia between 2015 and 2017 and were diagnosed with definite or probable ALS according to the revised El Escorial criteria.^38^ Control subjects were healthy individuals free of neuromuscular diseases, recruited as either partners or friends of patients with ALS or community volunteers. Those with a recorded family history for ALS (including those recorded as testing positive for known mutations) were excluded as both cases or controls in both AU1 and AU2. Additional controls for the AU2 cohort were monozygotic (MZ) twin pairs aged >65 years contributed from the Older Australian Twin Study (OATS)^39^ recruited at QIMR Berghofer Medical Research Institute, University of New South Wales and the University of Melbourne. Twin pair data helped in quality control (QC) checks but only one twin from each pair was used in analyses. Written consent was obtained from all individuals enrolled in this study, and the study was approved by the corresponding HREC at the different sites: University of Sydney, Western Sydney Local Health District, Royal Brisbane and Women Hospital Metro North, South Metropolitan Health Service, Macquarie University, QIMR Berghofer Medical Research Institute, University of New South Wales and the University of Melbourne. The mean predicted age,^40^ predicted smoking scores,^29^ and sex distribution between cases and controls for the cohorts used in this study can be found in Supplementary Table 5.

A cohort from the Netherlands (NL) was available to us for replication analyses, collected under Project MinE.^41^ The participants of this study consisted of 1866 Netherlands individuals (*N* = 1222 cases, *N* = 644 controls).^42^ All ALS cases were diagnosed with definite or probable ALS according to the revised El Escorial criteria,^38^ and those with a recorded family history for ALS were excluded. All participants gave written informed consent and the institutional review board of the University Medical Center Utrecht approved this study.

### DNA methylation data

For the AU ALS datasets, bisulfite conversions were performed in 96-well plates using the EZ-96 DNA Methylation Kit (Zymo Research, Irvine, CA, USA). Prior to conversion, DNA concentrations were determined by the Take3™ Micro-Volume Plate on the Epoch™ Microplate Spectrophotometer (BioTek Instruments, Inc.) and standardized to include 500 ng. Three technical replicates were included in each conversion to assess repeatability. The following DNA samples were obtained from the NIGMS Human Genetic Cell Repository at the Coriell Institute for Medical Research: CEPH NA06997 and CEPH NA07029. Each DNA sample was included as a control on alternating plates. One sample from each run was duplicated on the plate and one sample duplicated from a different plate. DNA recovery after conversion was quantified using the Take3™ Micro-Volume Plate on the Epoch™ Microplate Spectrophotometer (BioTek Instruments, Inc.). Samples that showed incomplete bisulfite conversion (calculated concentration <25 ng/μl) were not taken forward to the assessment of DNA methylation. Bisulfite converted DNA samples were hybridized to the 12 sample, Illumina Infinium HumanMethylation450 Beadchip (Illumina Inc., San Diego, CA) using the Infinium HD Methylation protocol and Tecan robotics. DNAm data for the NL sample were generated under similar protocols.

### QC and normalization of DNA methylation data

Data QC and normalization were conducted using the meffil R package.^43^ The same pipeline for DNA methylation (DNAm) data processing and QC was applied to all samples. AU1 and AU2 samples were processed together (along with additional samples from other studies). QC threshold parameters (Supplementary Note) determined samples and DNAm sites to exclude prior to normalization (Supplementary Table 6). Functional normalization (FN) was performed to remove technical variation, as described elsewhere.^44^ The most variable normalized probes (*m* = 20,000) were extracted, decomposed into principal components, and each component regressed against slide, chip column, chip row, and sex to test for batch effects. The association detection *p* value threshold was set to 0.01. For both datasets technical variation was not completely removed after FN and some known confounders affect DNA methylation. Thus, we perform an additional adjustment step using the normalized DNAm probe values (from meffil, as described above) in linear regression models as response variable and predicted age, sex, chip position, predicted CTP (excluding eosinophils, because of redundancy in proportion data), predicted smoking scores and slide (as random effect) as covariates. The residuals from this adjustment step were used for downstream MWAS analyses. We hypothesized the effect of this pre-adjustment should have a more pronounced effect in standard linear regression MWAS results compared to mixed linear models. Supplementary Fig. 6 supports this hypothesis, with a more pronounced reduction in significance of results in linear regression (Supplementary Fig. 6a) compared to both MOA and MOMENT (Supplementary Fig. 6b, c, respectively). We also observed a lower correlation of effect sizes (Supplementary Fig. 6d), between linear regression with pre-adjusted and non-adjusted DNA methylation values (${\hat{\mathrm r}}_{{\mathrm{b}}\_{\mathrm{Linear}}\_{\mathrm{adj}}} = 0.85$, s.e. = 6.7 × 10^−3^), compared to both MOA (${\hat{\mathrm r}}_{{\mathrm{b}}\_{\mathrm{MOA}}\_{\mathrm{adj}}} = 0.99$, s.e. = 1.6 × 10^−3^) and MOMENT (${\hat{\mathrm r}}_{{\mathrm{b}}\_{\mathrm{MOMENT}}\_{\mathrm{adj}}} = 0.97$, s.e. = 5.7 × 10^−3^, Supplementary Fig. 6e, f, respectively). Related individuals, sex-chromosome linked probes, probes influenced by SNPs, and probes with non-unique hybridization and extension were also removed prior to analysis, following recommendations described elsewhere.^45^ Afterwards, we removed remaining probes with s.d. <0.02. This decision is justified, because power to detect an association depends in part on the variance between individuals and (standardized) effect sizes. Excluding these DNAm sites also reduces the multiple testing burden in MWAS. In all, 160,304 probes remained for analysis in the AU cohort (Supplementary Table 6).

### Omics residual maximum likelihood analyses (OREML): variance captured by all DNAm sites

Based on extensive simulation and application to real DNAm data, Zhang et al.^18^ developed a method with one random-effect component to estimate the proportion of trait variance captured by all DNAm sites. The random-effect model can be written as

1 $${\mathbf{y}} = {\mathbf{C\beta }} + {\mathbf{Wu}} + {\mathbf{e}}\,{\mathrm{with}}\,{\mathrm{var}}\left( {\mathbf{y}} \right) = {\mathbf{V}} = {\mathbf{WW}}^\prime {\upsigma}_{\mathrm{u}}^2 + {\mathbf{I}}{\upsigma}_{\mathrm{e}}^2 = {\mathbf{A}}{\upsigma}_o^2 + {\mathbf{I}}{\upsigma}_{\mathrm{e}}^2,$$

where **y** is an *n* × 1 vector of phenotype values of *n* individuals, **C** is an *n* × *p* matrix of *p* covariates (e.g., age, sex, smoking status), **β** is a *p* × *1* vector of the fixed covariate effects on the phenotype, **W** is an *n* × *m* matrix of *m* standardized DNAm values, where *m* is the number of DNAm sites, **u**_*i*_ is an *m* × *1* vector of the joint random probe effects on the phenotype, and **e** is an *n* × *1* vector of residuals. The variance of **y** is var(**y**) = **V**=WW′$\sigma _{\mathrm{u}}^2 + {\mathbf{I}}\sigma _{\mathrm{e}}^2$ . We can re-write this equation as ${\mathbf{V}} = {\mathbf{A}}\sigma _{\mathrm o}^2 + {\mathbf{I}}\sigma _{\mathrm{e}}^2$ with **A** = **WW**′/*m* and $\sigma _o^2 = {\mathrm{m}}\sigma _u^2$, where **A** is then the omics-data-based relationship matrix (ORM) and $\sigma _u^2$ is the variance between individuals attributed to genome-wide DNAm differences. The variance components can be estimated by REML. The proportion of variance attributable to genome-wide DNAm, $\rho ^2 = \frac{{\sigma _o^2}}{{\sigma _o^2 + \sigma _e^2}}$, is similar to the SNP-based heritability concept in GREML,^24^ but where variances represent factors that may include both causes and consequences of the phenotype. The baseline variance of the phenotype case–control status in the AU sample is approximated as the binomial variance of $\hat \sigma _{\boldsymbol{P}}^2$ = *P*(1−*P*) = 0.246, where *P* is the proportion of the sample that are cases (*P* = 0.56), with the reported $\hat \sigma _{\boldsymbol{P}}^2$ being the sum of the estimates of the variance components $\hat \sigma _o^2 + \hat \sigma _e^2$.

### Linear regression DNA MWAS analysis

For linear regression MWAS we used models without:

2 $${\mathbf{y}} = {\mathbf{w}_{i}} {{b}_{i}} + {\mathbf{e}}$$

and with covariates

3 $${\mathbf{y}} = {\mathbf{w}_{i}} {{b}_{i}} + {\mathbf{C}} {\mathbf{\beta }} + {\mathbf{e}}$$

where **w**_*i*_ (a *n* × *1* vector of DNAm measures of a probe *i*, i.e., the target probe) and *b*_*i*_ (the effect of probe *i* on the phenotype; fixed effect). We used the 10 principal components estimated from the ORM as covariates.

### MLM-based omics association (MOA) and multi-component MLM-based omics association excluding the target (MOMENT) MWAS analyses

We conducted MLM MWAS using both MOA and MOMENT. The MOA MWAS model is

4 $${\mathbf{y}} = {\mathbf{w}_{i}} {{b}_{i}} + {\mathbf{Wu}} + {\mathbf{e}}$$

In this model, the probe being tested is fitted twice, once as a fixed and also as a random effect, which results in slightly reduced power compared to a (hypothetical) model in which the focal probe is excluded from **W**, but this would be computationally very demanding. In this model it is assumed that all probe effects follow a single distribution, which may not reflect the true distribution. In the MOMENT model^18^ DNAm probe effect sizes are drawn from two effect size distributions for different probes sets, selected according to their association statistics in an initial linear regression model, with each group then fitted as a random-effect:

5 $${\mathbf{y}} = {\mathbf{w}}_{i} {b}_{i} + \mathop {\sum }\limits_{{j}} {\mathbf{W}}_{{j}}{\mathbf{u}}_{{j}} + {\mathbf{e}}$$

where **W**_j_ is an *n x m*_*j*_ matrix of standardized DNAm probe values in the *j*th group, and *m*_*j*_ is the number of DNAm sites in the group (excluding the DNAm sites in the 100 Kb region centred at the probe being tested). After conducting MWAS, DNAm sites were mapped to the latest GRCh38/hg38 genome build^45^ and annotated to genes, based on GENCODE v22. We used the *r*_b_ method^25^ to quantify the similarity between probe effects, which accounts for sample overlap and errors in the estimated probe effects.

### Out-of-sample classification using DNA MPSs

MPS were calculated for each individual in the NL sample as the sum of DNAm probe values weighted by their effect sizes estimated in the AU sample (Supplementary Fig. 3). In different analyses DNAm probe effect sizes were estimated from the linear regression, MOA or MOMENT MWAS or the BLUP-estimated probe values of **u** from Eq. (1), as implemented in OSCA.^18^ In the BLUP models, predicted age, sex, predicted smoking scores, and batch effects (chip position and slide) were fitted as fixed effects. Classification efficacy of the MPS was evaluated by the area under the receiver-operator characteristic curve (AUC) that relates the false-positive rate (specificity) to the true-positive rate (sensitivity), in logistic regression. We used the R package pROC^46^ to plot the receiver-operator characteristic curves and calculate AUC for each MPS. To evaluate the possible gain in classification accuracy, we calculated MPS from the fixed effects of predicted CTP, estimated in an OREML analysis (Eq. (1)). Analyses were conducted in R version 3.5.0 and OSCA v0.45.

### Reporting summary

Further information on research design is available in the Nature Research Reporting Summary linked to this article.

## Supplementary information

Supplementary Information

Reporting summary

## Data Availability

The Australian data are available from the author upon reasonable request; a dbGAP submission for upload of the Australian data is in progress. The Netherlands dataset was available to us for replication purposes.

## Code availability

The code used to analyze the data and to generate the figures in this manuscript can be found at https://github.com/m-nabais/201909_als_dnam_manuscript.
