# Supplementary material for: Significant out-of-sample classification from methylation profile scoring for amyotrophic lateral sclerosis
Source: NPJ Genom Med. 2020 Feb 27;5:10. doi: 10.1038/s41525-020-0118-3 (PMC7046630; doi:10.1038/s41525-020-0118-3)
Supplement: Supplementary file 1 — Supplementary Information [file 41525_2020_118_MOESM1_ESM.pdf]

SUPPLEMENTARY INFORMATION

Significant out-of-sample classification from methylation profile scoring for amyotrophic lateral sclerosis

Nabais *et al*

Table of Contents

Comparing effect sizes and significance of results between linear and mixed linear models regression *2*

Supplementary Figure 1 2

Post-hoc power analyses in replication cohort *3*

Supplementary Figure 2 3

Out-of-sample classification results *4*

Supplementary Figure 3 4

Supplementary Figure 4 5

Supplementary Table 1 6

Supplementary Figure 5 7

Supplementary Table 2 8

Supplementary Table 3 9

Cohort descriptions *10*

Supplementary Table 4 10

Supplementary Table 5 10

Effects of pre-adjusting DNAm probes have a more pronounced effect in standard linear regression MWAS results compared to mixed linear models *11*

Supplementary Figure 6 11

Supplementary Note - Literature evidence of a functional role of CXXC5 and MOMENT 25 most associated DNAm sites *12*

Functional annotation of MOMENT 25 most associated DNAm sites 12

Supplementary Figure 7 14

Annotation of top 25 MOMENT probes to genes: literature evidence in humans and animal models of ALS 14

CXXC5 and neurodegeneration 16

Supplementary Note - QC parameters for exclusion of samples and probes *17*

Supplementary Table 6 18

References *19*

Comparing effect sizes and significance of results between linear and mixed linear models regression

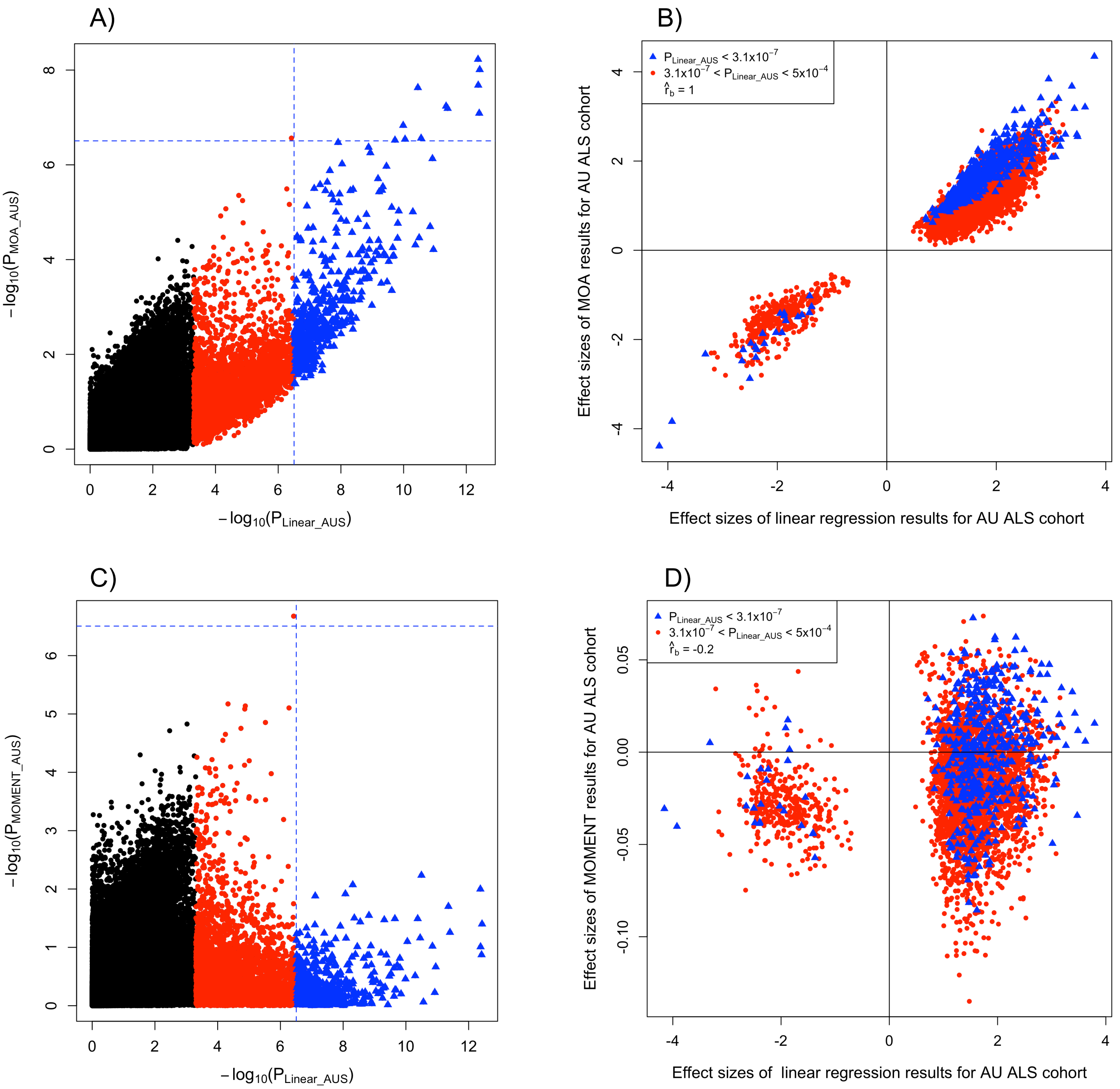

Supplementary Figure 1 - Correlation of effect sizes between linear regression and mixed linear model MWAS, in the Australian ALS cohort. A) -log10(p) of all probes in linear regression (x-axis) and MOA (y-axis), for the AU ALS dataset. **B)** Effect sizes of linear regression (x-axis) and MOA (y-axis), for AU ALS dataset, of probes with $p < 5 \times 10^{-4}$ from linear regression. Correlation of effect sizes between linear regression and MOA results: $\hat{r}_b = 1$, s.e. = 3×10^{-3}. **C)** log10(p) of all probes in linear regression (x-axis) and MOMENT (y-axis), for the AU ALS dataset. **D)** Effect sizes of linear regression (x-axis) and MOMENT (y-axis), for AU ALS dataset, of probes with $p < 5 \times 10^{-4}$ from linear regression. Correlation of effect sizes between linear regression and MOMENT results: : $\hat{r}_b = -0.2$, s.e. = 0.02. Dashed blue lines in **A)** and **C)** mark the genome-wide significance threshold ($p = 3.1 \times 10^{-7}$) of linear regression, MOA and MOMENT. Red dots mark all probes with $p < 5 \times 10^{-4}$ from linear regression (m = 3,596) as in **B)** and **D)**.

Post-hoc power analyses in replication cohort

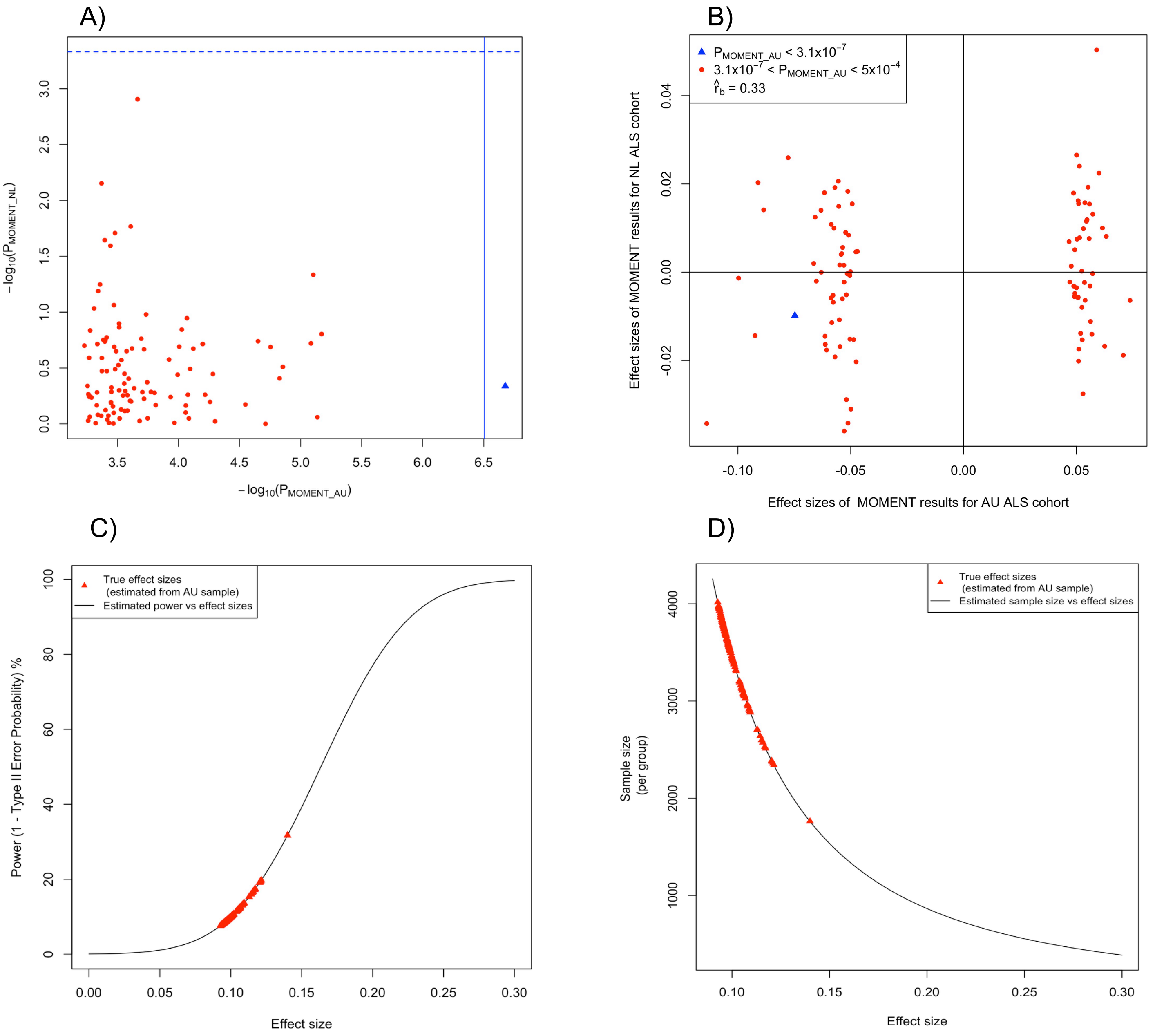

Supplementary Figure 2 - MWAS MOMENT results of probes with p-value < 5x10^{-5}, from the AU ALS cohort, are not replicated in an independent Netherlands (NL) ALS cohort. A) -log10(p-value) of probes with p-value < 5x10^{-4} in MOMENT (x-axis) and corresponding p-values of MOMENT analysis of NL ALS replication cohort (y-axis). Solid blue line marks the genome-wide significance threshold (p-value = 3.1x10^{-7}) of AU MOMENT MWAS. Dashed blue line marks replication significance threshold of NL MOMENT MWAS (p-value = 0.05/94, i.e, 5.3x10^{-4}). Blue triangle marks the genome-wide significant probe in AU MOMENT, as in **B)** Effect sizes of MOMENT (x-axis) probes with p-value < 5x10^{-4}, from the AU ALS cohort and corresponding effect sizes of MOMENT MWAS of the NL ALS replication cohort (y-axis). Correlation of effect sizes: $\hat{r}_b$ = 0.33, se = 0.2. **C)** Post-hoc statistical power calculation, based on the pre-determined NL cohort sample size (N_{cases} = 1159, $N_{controls}$ = 637) and replication significance threshold p-value = 5.3x10^{-4}. Power is calculated as a percentage of 1 - probability of a type II error (y-axis) and is plotted with estimated effect sizes (x-axis). Red triangles represent the true effect sizes of probes with p-value < 5x10^{-4} in MOMENT in common between datasets (m = 97), calculated from the AU sample. **D)** Post-hoc statistical calculation of sample size (y-axis) necessary to find a true association with the corresponding effect sizes (x-axis), based on pre-determined 80% power and replication significance threshold p-value = 5.3x10^{-4}.

Out-of-sample classification results

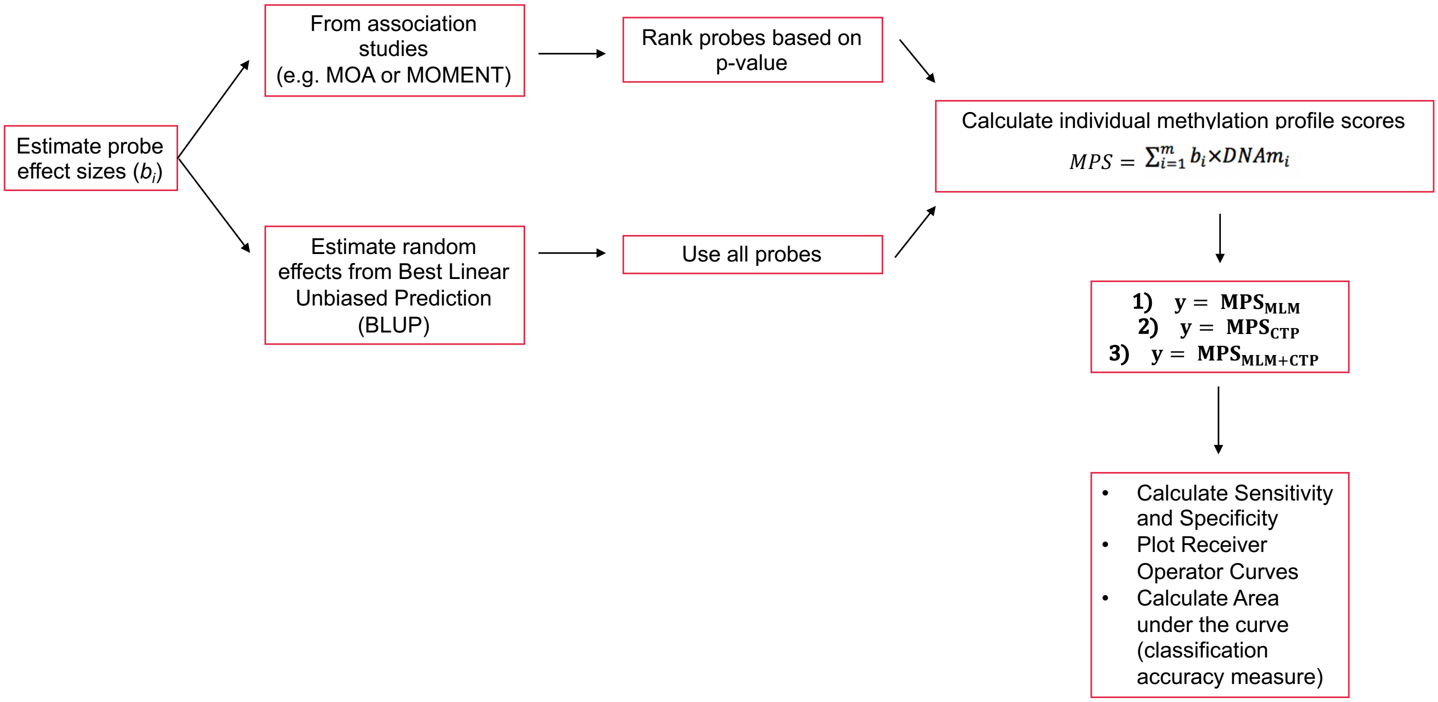

Supplementary Figure 3 - Schematic representation of out-of-sample classification using methylation profile scores (MPS). An MPS is calculated for each individual in the target sample as the sum of DNAm probe values multiplied by their effect sizes, estimated in a discovery sample. In different analyses effect sizes are estimated from MOA or MOMENT MWAS or BLUP. In the BLUP MLM predicted age, sex, predicted smoking scores and batch effects (chip position and slide number) were fitted as fixed effects. Classification efficacy of the MPS was evaluated by the area under the receiver-operator characteristic curve (AUC) that relates false positive rate (Specificity) vs true positive rate (Sensitivity) in logistic regression. To evaluate the possible gain in classification accuracy, we also used the estimated fixed effects of predicted cell proportions (CTP) when calculating MPS. These were estimated using an OREML model, with case-control status as the response variable and predicted cell proportions (excluding Eosinophils), as independent variables (see Methods). We made classifications from AU as the discovery sample and NL as the target sample. MPS_{MLM} - MPS derived from BLUP or MOA or MOMENT effect sizes; MPS_{CTP} - MPS derived from predicted CTP effect sizes. $MPS_{MLM+CTP}$ - MPS derived from BLUP or MOMENT effect sizes combined with predicted CTP effect sizes.

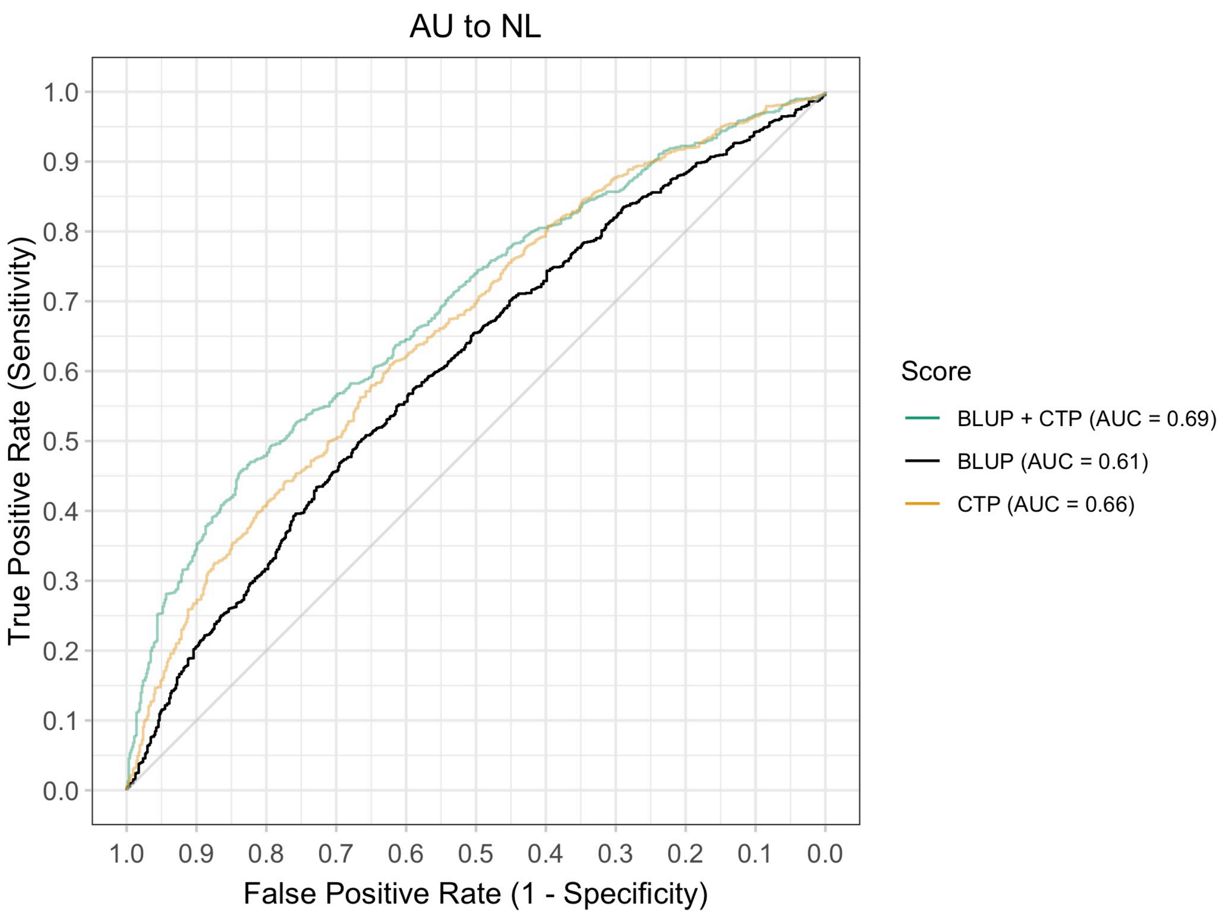

Supplementary Figure 4 - Receiver operator characteristic (ROC) curves and area under the curve (AUC), based on ALS-derived methylation BLUP scores with AU as discovery cohort (N = 1395) and NL as validation cohort (N = 1796). ROC curves were plotted from Specificity vs Sensitivity values for classifying an individual as case or control, at different thresholds of methylation profile scores. The AUCs were then calculated for each ROC curve. **Black** - classification accuracy calculated from methylation profile scores based on BLUP solutions of methylation probes only; orange – classification accuracy calculated from methylation profile scores based on estimated fixed effects of predicted cell type proportions (CTP) only; green – classification accuracy calculated from methylation profile scores based on the sum of the scores from BLUP and CTP. The combined BLUP+CTP score gives equal weight to the two contributing scores. It may be more optimal to give unequal weight to the two scores, but another independent data is needed to estimate these weights.

Supplementary Table 1 - Area under the curve (AUC) of ALS-derived methylation profile scores based on p-value thresholding from AU MOA or AU MOMENT to an independent ALS cohort from the Netherlands. The p-value (P) is from logistic regression models. m - number of probes, CI95% - confidence interval at 95% level for AUC, P - p-values from logistic regression models.

	**MOA AUC**	**m**	**CI 95%**	**P**	**MOMENT AUC**	**m**	**CI 95%**	**P**	**Linear regression (No covariates) AUC**	**m**	**CI 95%**	**P**	**Linear regression (With PCs) AUC**	**m**	**CI 95%**	**P**
$p < 0.5$	0.6	74,556	[0.57-0.63]	2.7x10^{-4}	0.61	75,048	[0.59-0.64]	6.2x10^{-10}	0.61	79,993	[0.59-0.64]	4.8x10^{-6}	0.61	77,479	[0.58-0.64]	9.9x10^{-16}
$p < 0.2$	0.57	30,443	[0.54-0.59]	0.06	0.62	30,334	[0.59-0.65]	2.3x10^{-10}	0.59	37,880	[0.57-0.62]	7.1x10^{-3}	0.59	33,210	[0.56-0.62]	9.9x10^{-12}
$p < 0.1$	0.54	15,559	[0.51-0.57]	0.38	0.62	15,211	[0.59-0.65]	3.7x10^{-11}	0.57	23,103	[0.54-0.59]	0.27	0.57	17,804	[0.54-0.59]	3.3x10^{-8}
p < 1x10^{-2}	0.51	1,940	[0.48-0.54]	0.23	0.64	1,565	[0.61-0.67]	9.3x10^{-15}	0.52	7,223	[0.49-0.54]	0.08	0.48	2,543	[0.45-0.51]	0.05
p < 1x10^{-3}	0.55	343	[0.52-0.57]	6.6x10^{-3}	0.64	179	[0.62-0.67]	3.7x10^{-17}	0.54	3,877	[0.51-0.57]	0.01	0.52	501	[0.49-0.55]	0.36
p < 1x10^{-4}	0.54	102	[0.51-0.57]	0.02	0.65	25	[0.62-0.68]	8.3x10^{-22}	0.55	2,392	[0.52-0.57]	6.5x10^{-3}	0.54	165	[0.51-0.57]	0.03
p < 1x10^{-5}	0.56	39	[0.53-0.59]	1.1x10^{-3}	0.55	5	[0.52-0.58]	6.5x10^{-4}	0.55	1,410	[0.52-0.58]	3.8x10^{-3}	0.55	76	[0.52-0.58]	4.4x10^{-3}
p < 3.1x10^{-7} (MWAS genome-wide significance threshold)	0.55	10	[0.52-0.58]	1.1x10^{-3}	0.54	1	[0.51-0.56]	0.02	0.55	462	[0.52-0.58]	1.6x10^{-3}	0.56	25	[0.53-0.59]	3.2x10^{-4}

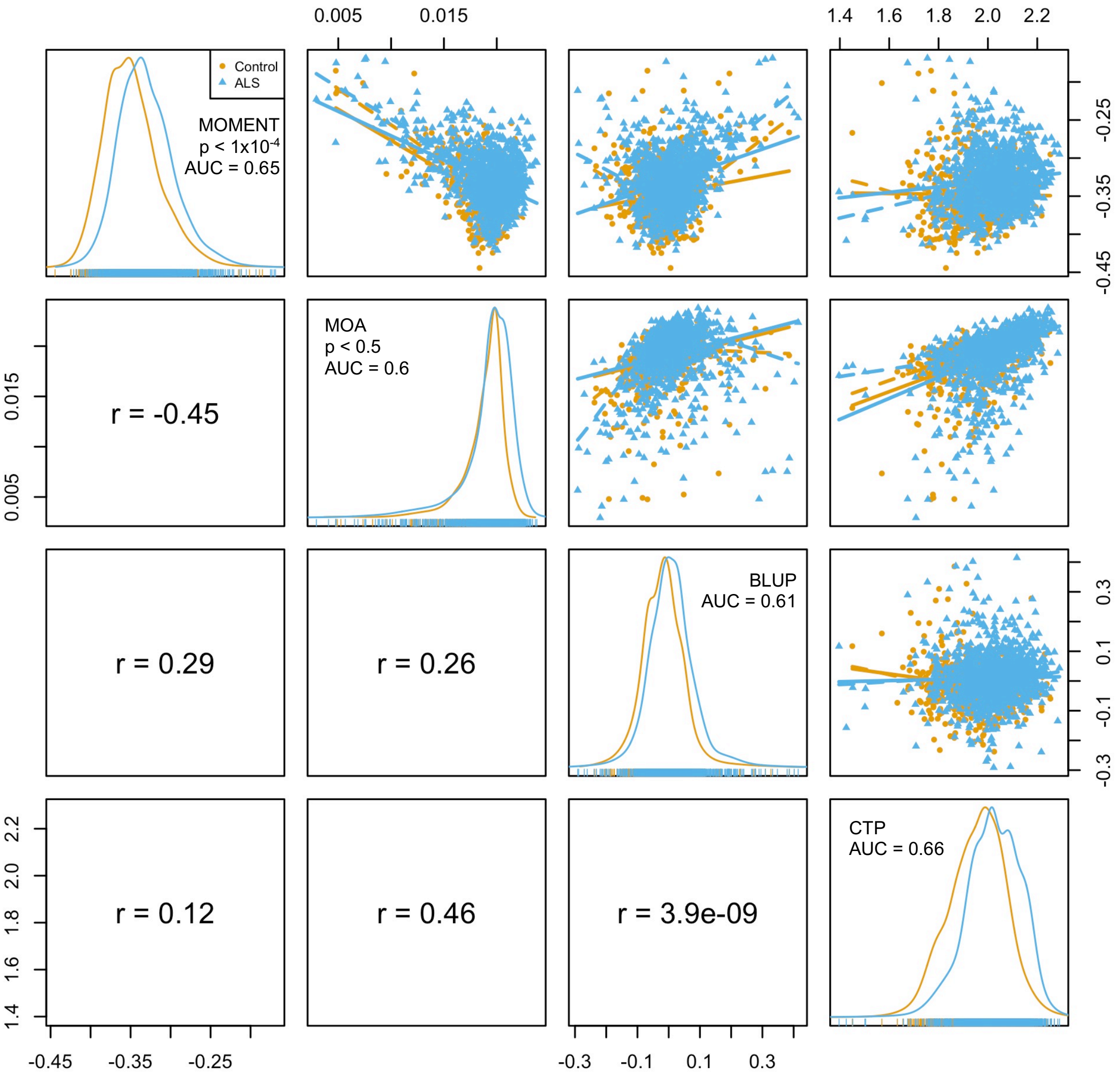

Supplementary Figure 5 - Scatter matrix and correlation of individual methylation profile scores (MPS) based on estimated effect sizes of DNA methylation sites, from different mixed-linear model (MLM) methods and effect sizes of cell-type proportions, excluding eosinophils. The latter were estimated from an OREML model. CTP - cell-type proportions.

Supplementary Table 2 - DNA methylation DNAm sites with $p < 1x10^{-4}$ using the combined Australian cohort in MOMENT. Chr – chromosome number, Probe – probe identification number as provided by Illumina, bp – base pair position in the genome, Gene – closest genes the probe is annotated to, based on distance to transcription starting site, Orientation – DNA strand orientation (F = forward, R = Reverse), b_MOMENT – effects sizes (increase (positive sign) or decrease (negative sign) of methylation between cases and controls per standard deviation unit) of AU MOMENT, p_MOMENT – p-values of AU MOMENT.

Chr	Probe	bp	Gene	Orientation	b_MOMENT	p_MOMENT
5	cg04104695	139679164	CXXC5	R	-0.14	$2.1x10^{-7}$
3	cg18670076	189957373	P3H2/LEPREL1	F	0.12	$6.7x10^{-6}$
1	cg07181952	1304619	ACAP3	R	-0.12	$7.2x10^{-6}$
1	cg17901584	54888033	RP11-67L3.4;DHCR24	R	0.12	$7.8x10^{-6}$
2	cg24166814	55840142	NA	R	-0.12	$8.2x10^{-6}$
21	cg16081992	33560141	SON;DONSON	F	-0.11	$1.3x10^{-5}$
6	cg18880660	6321899	F13A1	F	0.11	$1.4x10^{-5}$
2	cg04229930	223569118	NA	R	0.11	$1.7x10^{-5}$
3	cg06059360	42616126	RP4-613B23.1;NKTR	F	-0.11	$1.9x10^{-5}$
2	cg03785076	240997498	SNED1	F	-0.11	$2.2x10^{-5}$
4	cg02846963	181509662	NA	R	0.11	$2.8x10^{-5}$
3	cg22509807	180152574	NA	F	-0.10	$5.0x10^{-5}$
12	cg10301695	6124551	VWF	R	-0.10	$5.2x10^{-5}$
1	cg20741620	1036845	AGRN	F	-0.10	$5.5x10^{-5}$
11	cg06365843	41308796	LRRC4C	F	0.10	$6.1x10^{-5}$
22	cg22650271	39364160	SYNGR1	R	-0.10	$6.3x10^{-5}$
15	cg05110803	98842094	IGF1R	F	-0.10	$7.5x10^{-5}$
16	cg04708264	87289814	RP11-178L8.5;C16orf95	R	0.10	$8.1x10^{-5}$
3	cg05709770	59717843	NA	F	-0.10	$8.2x10^{-5}$
5	cg13897374	150625221	SYNPO	R	-0.10	$8.3x10^{-5}$
2	cg04915300	156458413	GPD2	R	0.10	$8.5x10^{-5}$
3	cg01966117	52494698	STAB1	R	-0.10	$8.7x10^{-5}$
9	cg13444518	132591386	NA	F	0.10	$8.7x10^{-5}$
6	cg03546163	35686586	FKBP5	F	-0.10	$9.4x10^{-5}$
8	cg14195992	47353350	SPIDR	F	-0.10	$9.8x10^{-5}$

Supplementary Table 3 - Top-most associated mQTL SNPs for the top-most significantly associated MOMENT DNAm sites and their corresponding p-values in the largest GWAS of ALS to date. mQTL SNPs were associated with 9 of the 25 DNAm probes. The most associated SNP with the top MOMENT probe cg04104695 in the brain mQTL meta-analysis was rs12108986 ($p = 7.4x10^{-8}$). None of the mQTLs overlapped with significant SNPs from the published GWAS based on 20,806 cases and 59,804 controls, and hence we have no evidence for overlap between the MWAS and GWAS results. SNP - single nucleotide polymorphism identifier, Chr - chromosome, BP - SNP base pair position, A1 - mQTL effect allele, A2 - mQTL alternate allele, Freq - allelic frequency of effect allele, Orient – DNA strand orientation, F = forward, R = Reverse.

SNP	Chr	BP	A1	A2	Freq	Probe	Probe_bp	Gene	Orient	b mQTL	s.e. mQTL	p mQTL	p ALS GWAS	p ALS MOMENT
rs1666426	3	189664326	G	C	0.56	cg18670076	189957373	P3H2/ LEPREL1	F	-0.61	0.06	$1.03x10^{-26}$	0.58	$5.9x10^{-6}$
rs253347	5	150005206	C	T	0.49	cg13897374	150625221	SYNPO	R	0.55	0.06	$7.6x10^{-21}$	0.34	$8.3x10^{-5}$
rs306534	9	135515544	T	C	0.59	cg13444518	132591386	-	F	0.52	0.06	$9.3x10^{-18}$	0.58	$7.3x10^{-5}$
rs58799742	22	39767390	G	A	0.01	cg22650271	39364160	SYNGR1	F	0.78	0.09	$2.3x10^{-17}$	0.47	$9.1x10^{-5}$
rs72807735	2	56066916	A	G	0.93	cg24166814	55840142	-	R	0.55	0.07	$1.6x10^{-15}$	0.48	$1.2x10^{-5}$
**rs12108986**	**5**	**139068126**	**C**	**G**	**0.66**	**cg04104695**	**139679164**	**CXXC5**	**F**	**-0.24**	**0.04**	$\mathbf{7.4x10^{-8}}$	**0.03**	$\mathbf{2.3x10^{-7}}$
rs729210	11	41259180	C	T	0.79	cg06365843	41308796	LRRC4C	R	-0.41	0.07	$9.3x10^{-8}$	0.93	$5.1x10^{-5}$
rs2590838	3	52622086	A	G	0.51	cg01966117	52494698	STAB1	F	0.28	0.06	$3.9x10^{-6}$	0.73	$8.7x10^{-5}$
rs2309504	4	182482693	T	C	0.41	cg02846963	181509662	-	F	0.26	0.06	$2.9x10^{-5}$	0.14	$2.8x10^{-5}$

Cohort descriptions

Supplementary Table 4 - Summary of ALS cases and controls distribution between sites of origin. AU1 is composed of samples originating from MND DNA Bank (NSW), after quality control. AU2 is composed of all other samples, after quality control. VIC - Victoria, WA - Western Australia, NSW - New South Wales, QLD - Queensland.

Site of Origin	Cases	Controls
AU1: MND DNA Bank	440	418
AU2:		
Calvary Health Care Bethlehem (VIC)	24	0
Fiona Stanley Hospital (WA)	9	0
Macquarie University Multidisciplinary Motor Neurone Disease Clinic (NSW)	141	50
Older Australian Twins Study OATS	0	84
Royal Brisbane Women's Hospital (QLD)	168	61
Total AU2	342	195
Total AU1+AU2	782	613

Supplementary Table 5 - Descriptive statistics for predicted age, smoking score and gender of cases and controls in the Australian and Netherlands ALS-control cohorts.

	AU		Netherlands	
	Control (n=613)	ALS (n=782)	Control (n=637)	ALS (n=1159)
**Predicted Age**				
Mean (SD)	60.4<br>-12.3	62.9<br>-11.4	68<br>-8.69	69.2<br>-9.16
**Predicted smoking score**				
Mean (SD)	3.58<br>-0.77	3.55<br>-0.78	3.67<br>-0.95	3.8<br>-1.07
**Gender**				
Male	280 (46%)	478 (61%)	272 (43%)	497 (43%)
Female	333 (54%)	304 (39%)	365 (57%)	662 (57%)

Effects of pre-adjusting DNAm probes have a more pronounced effect in standard linear regression MWAS results compared to mixed linear models

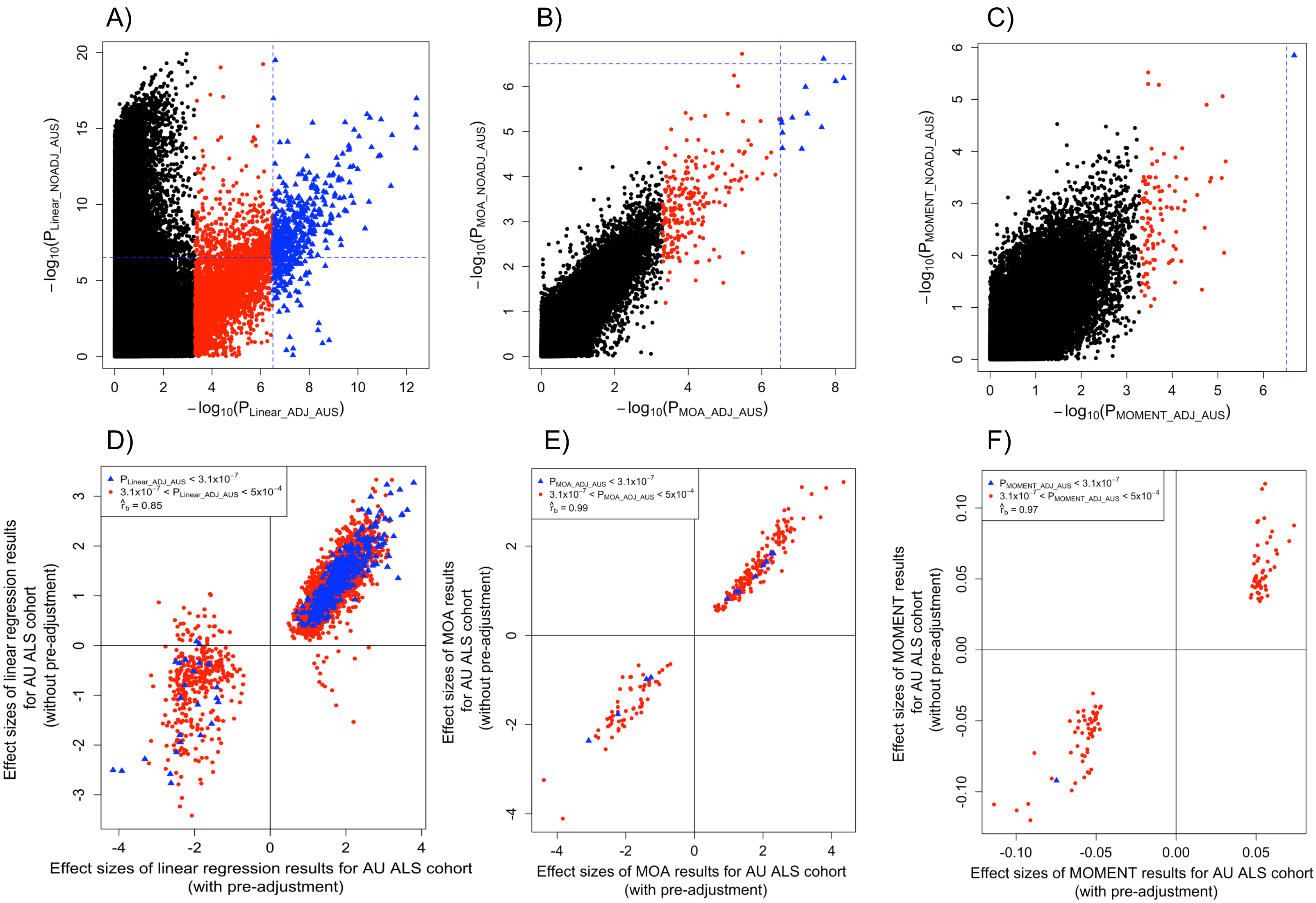

Supplementary Figure 6 - Comparison of MWAS results from linear and mixed linear model regression, for AU ALS cohort before and after pre-adjustment of DNA methylation probes with technical and biological covariates. A), B) and C) -log10(p) of all probes in linear regression, MOA and MOMENT, respectively, before (y-axis) and after (x-axis) pre-adjustment, for the AU ALS dataset. Dashed blue lines mark the genome-wide significance threshold ($p = 3.1\text{x}10^{-7}$). Red dots mark all probes with $p < 5\text{x}10^{-4}$ as in **D), E) and F)** Effect sizes of linear regression, MOA and MOMENT, respectively, before (y-axis) and after (x-axis) pre-adjustment, for the AU ALS dataset., of probes with $p < 5\text{x}10^{-4}$. Correlation of effect sizes: $\hat{r}_{b\_Linear\_adj}$ = 0.85, se = $6.7\text{x}10^{-3}$, $\hat{r}_{b\_MOA\_adj}$= 0.99, se = $1.6\text{x}10^{-3}$ and $\hat{r}_{b\_MOMENT\_adj}$ = 0.97, se = $5.7\text{x}10^{-3}$.

Supplementary Note - Literature evidence of a functional role of *CXXC5* and MOMENT 25 most associated DNAm sites

Functional annotation of MOMENT 25 most associated DNAm sites

Briefly, we used the R package *annotatr* [1] which provides genomic annotations and a set of functions to read, intersect, summarize and to visualize genomic regions in the context of genomic annotations. The basis for CpG related annotations are CpG islands (CGIs) tracks from the R package *AnnotationHub* (including CGIs, CGI shores and CGI shelves) and the basis for annotations related to genic features is the *TxDb.Hsapiens.UCSC.hg38.knownGene* object, of Homo sapiens data from University of California Santa Cruz build hg38 based on the knownGene Track (including 1-5kb upstream of a transcription starting site (TSS), promoters (< 1kb from TSS), 5 untranslated regions (UTRs), conding sequence, exons, first exons, introns, intron/exon and exon/intron boundaries, 3UTRs, and intergenic). When more than one transcript was present per gene, we kept the longer transcript.

Eight out of the top 25 most associated probes in MOMENT ($p < 1x10^{-4}$) had CpG and genic related co-annotations (Supplementary Figure 7). The majority of probes were annotated to CpG shores (< 2kb flanking CGI), one to a CpG shelf (<2kb flanking outwards from a CpG shore) and one to a CGI. Most DNAm studies studies of methylation in disease have typically focused on the functional importance of DNAm in promoters, motivated by the finding of transcriptional silencing of tumour-suppressor genes by CGI-promoter hypermethylation [2]. However, CGIs have been shown to be less dynamic and less variant in terms of methylation status, when probed across a variety of tissues and cell populations [3, 4]. On the contrary, most tissue- and cell-specific DNAm occurs at CpG shores. Similarly, most DNAm alterations in colon cancer was shown to not involve CGIs, but CpG shores [3]. These alterations were shown to have an inverse relationship with gene expression of associated genes, and they apply to shores located within 2 kb of an annotated transcriptional start site, but leave open the possibility of additional regulatory function for shores located in intragenic regions or gene deserts [3].

Aditionally, four of the eight probes with co-annotations were annotated to intronic regions (Supplementary Figure 7), three of which were located in CpG shores (annotated to CXXC5, FKBP5 and AGRN) and one in a CGI (annotated to SYNPO). DNA methylation

of intragenic regions is also correlated with higher levels of gene transcription and may be a mechanism that regulates the use of alternative promoters [5, 6]. Almost all these eight DNAm sites show a tendency for decreased methylation levels in patients, compared to controls. This parallels with genomes of cancer cells that tend to show global hypomethylation (alongside hypermethylation of the gene promoters). As Shenker & Flanangan postulate, “the consequences of hypomethylation, whether intergenic or intragenic, are also unclear, but may act in a similar manner to that postulated in plant intragenic sequences, where demethylation leads to the reactivation of repressed repetitive elements, and potentially further genomic instability” [6], a phenomenon clearly implicated in disease. Although we were unable to perform functional enrichment tests in

regulatory elements due to the small number of DNAm sites identified in our analyses, these results are congruent with the literature and thus point for a potentially relevant functional role of these DNAm sites, but we are wary of overinterpretation.

Annotation of top 25 MOMENT probes to genes: literature evidence in humans and animal models of ALS

The possibility that ALS may be a result of synaptopathy within the neuromotor system has been intensively researched in rodent models and humans and exhaustively reviewed elsewhere [7, 8]. This theory is compatible with glutamate excitotoxicity in ALS, the accumulation of misfolded proteins and mitochondrial dysfuncion at distal axons (corticospinal motorneurons, motor neurons and neuromuscular junctions, CSMN, MN and NMJ, respectively). It does not

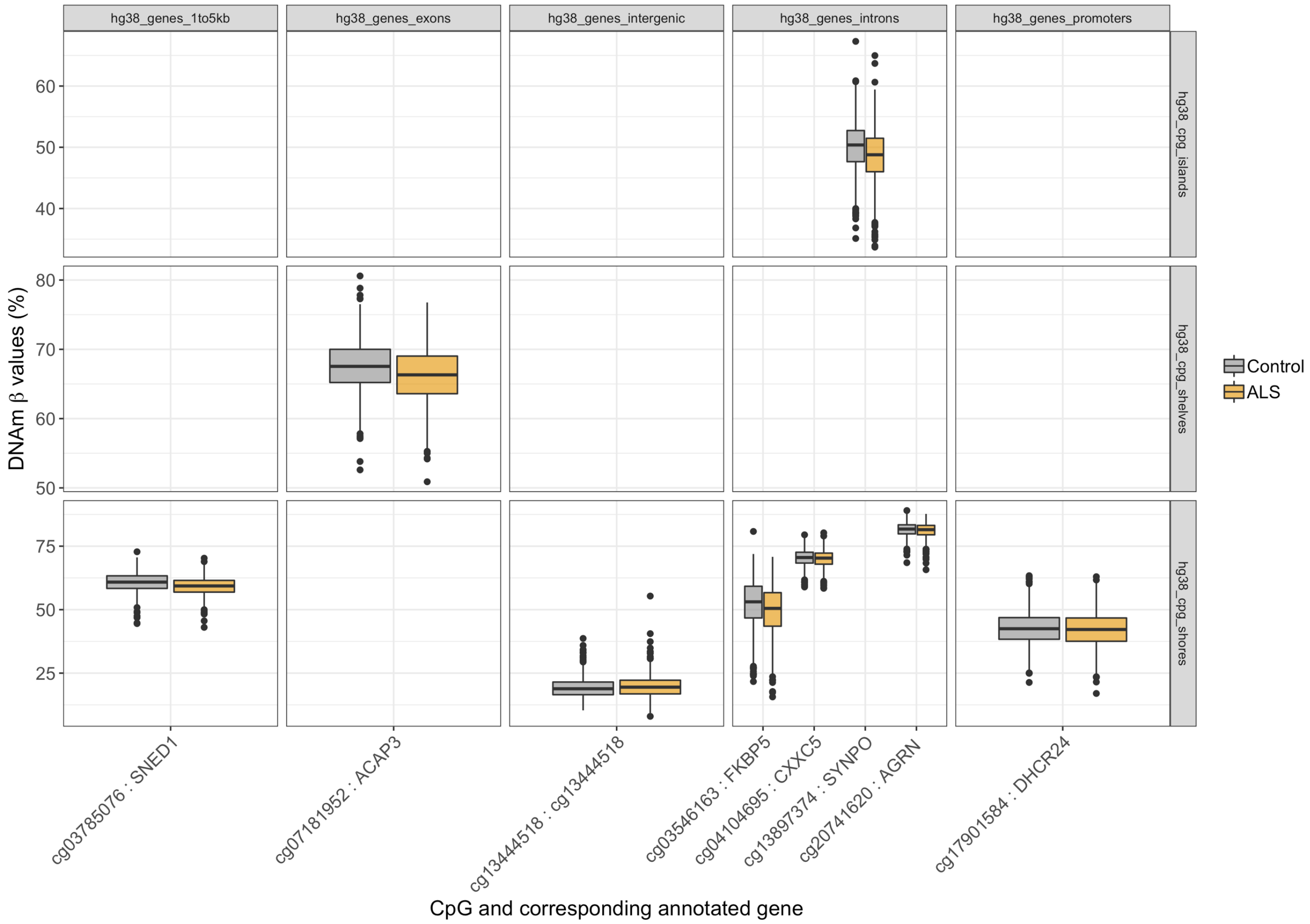

Supplementary Figure 7 - Boxplots of DNA methylation β values (%) of eight of the top 25 most associated probes in MOMENT that showed co-annotation of genic and CpG related features. DNAm sites are faceted across genic features annotations (columns) and CpG related features (rows). 1to5kb - 1 to 5kb of transcription starting site.

however, explain the contribution of neuroinflammation to the disease process, explain the initial protein misfolding, account for non-cell-autonomous influences or some of the muscle-specific modifiers of disease progression [9]. Indeed, due to the clear multifactorial nature of ALS and extensive pleiotropic effects of disease-altering variants [10, 11], one should be wary that some disease-related tissue abnormalities can interfere with the potential therapeutic properties. For example, AGRN has been extensively studied in the context of the NMJ, where the encoded protein exerts a key role as regulator of postsynaptic differentiation [12]. Overexpressing AGRN (and other genes involved in NMJ formation) in mouse models of ALS (and other neuromuscular disorders) has been shown to have therapeutic benefits [12]. However, in the context of the immune system, AGRN expression (and many other genes) was found to be associated with activation in monocytes from rapidly progressing ALS patients [13]. As another case example, the insulin-like growth-factor 1, IGF-1 is a well-studied trophic factor for different tissues, including nervous system and skeletal muscle. Animal studies have shown that $hSOD1^{G93A}$ mice at "end stage of disease" had normal levels of muscle IGF-I protein expression, but decreased circulating levels of IGF-I and skeletal muscle IGF-IRα protein expression [14, 15]. More importantly, these changes were variable according to disease progression. Interestingly, IGF-I-directed interventions prolong survival in a mouse model of ALS [16-18], whereas Growth-Hormone/IGF-I therapies were of no benefit in slowing disease progression in human ALS patients [19-21]. These seemingly inconsistent results illustrate the importance of acknowledging species-specific differences in disease progression and disease stage-specific or tissue-specific interventions, that may account for improved outcome in mouse models of disease.

Protein misfolding is a key feature of all neurodegenerative disorders, including alpha-synuclein in Parkinson's disease, tau and Aβ in Alzheimer's disease (AD), prion protein in prion diseases, polyglutamine disease proteins in polyglutamine repeat diseases (e.g., huntingtin in Huntington's disease), and SOD1 in amyotrophic lateral sclerosis. Peptidyl-prolyl cis/trans isomerases (PPIases), a unique family of molecular chaperones, regulate protein folding at proline residues. Similarly to the examples given above, some members of the PPIase family have been shown to exert positive and others negative effects on neuron function [22]. For example, FKBP5 is a PPIase, which acts as a co-chaperone that modulates not only glucocorticoid receptor activity in response to stressors [23], but has also been implicated in AD [24], showing neurotoxic effects. Interestingly, it was also shown to be overexpressed in monocytes of ALS patients compared to controls [13], once again highlighting the potentially widespread pleiotropic gene effects in complex disease traits.

CXXC5 and neurodegeneration

Genomic variation in the *CXXC5* locus has not been identified as contributing to ALS risk but it may play a relevant functional role. *CXXC5* encodes a retinoid inducible transcription factor containing a CXXC-type zinc finger motif. In the mature central nervous system (CNS), retinoids have been implicated in the maintenance of plasticity and neural stem cell production [25]. Retinoids can be converted into various retinoid species, including retinoic acid (RA) or retinol molecules, which are able to enter the cell. Once within the cell, RA can bind to a family of retinol-specific binding proteins, the cellular acid retinoid binding proteins, which are involved in the metabolism and nuclear import of RA [26, 27]. Elevated RA signaling in the adult correlates with axon outgrowth and nerve regeneration and has also been shown to be involved in the maintenance of the differentiated state of adult neurons [25]. Interestingly, it has been reported that disruption of RA signaling may also lead to degeneration of motor neurons [28], all relevant processes in ALS pathogenesis. *CXXC5* is ubiquitously expressed throughout human tissues, with relatively high expression in the brain. Current human exome and genome data suggests an essential functional role for *CXXC5* as the observed loss of function variation is lower than expected (pLI = 0.89, gnomAD). This may be due to its role in the CNS, which has been extensively studied in mouse models, where *CXXC5* was characterized as BMP4-regulated modulator of Wnt signaling in neural stem cells and also an important myelination factor, controlling multiple genes involved in myelination in oligodendrocytes [29, 30]. Moreover, a recent study has shown that ALS-patient derived oligodendrocytes (from both induced pluripotent stem cells and induced neural progenitor cells) have recently been found to play an active role in motor neuron death [31]. Thus, further research may be warranted to better understand the putative role between *CXXC5* and neurodegeneration in the context of ALS.

Supplementary Note - QC parameters for exclusion of samples and probes.

1. Exclude DNAm sites with low bead numbers in high proportion of samples (proportion of samples with bead number < 3 is > 0.1);
2. Exclude DNAm sites with only background signal in high proportion of samples (proportion of samples with detection p > 0.01 is > 0.1);
3. Exclude samples with high proportion of undetected DNAm sites (proportion of DNAm sites with detection p > 0.01 is > 0.1);
4. Exclude samples with high proportion of DNAm sites with low bead number (proportion of DNAm sites with bead number < 3 is > 0.1);
5. Regress median methylated signal on median unmethylated signal and exclude samples whose median methylated signal exceeded 3 standard deviations (SD) from the predicted values;
6. Exclude samples whose control DNAm sites mean value exceeded 5 SD from the mean across all DNAm sites;
7. The median intensity methylated vs unmethylated signal for all control DNAm sites exceeded 3 SD;
8. Calculate difference between median chromosome Y and chromosome X probe intensities ("XY diff"). Cutoff for sex differentiation was "XY diff" = -2. Exclude samples whose XY diff is higher than std = 5 (sex outliers);

Supplementary Table 6 - Number of individuals and DNAm probes passing QC and used for MWAS, OREML and out-of-sample classification analyses.

	AU	Netherlands
Individuals passing QC	1395	1796
Autosomal probes passing QC	445,194	415,126
Probes not cross-hybridizing and no SNP, with standard deviation > 0.02	160,304 (AU)	209,576

References

1. Cavalcante RG, Sartor MA: **annotatr: genomic regions in context**. *Bioinformatics* 2017, **33**(15):2381-2383.
2. Jones PA, Laird PW: **Cancer-epigenetics comes of age**. *Nature Genetics* 1999, **21**(2):163-167.
3. Irizarry RA, Ladd-Acosta C, Wen B, Wu Z, Montano C, Onyango P, Cui H, Gabo K, Rongione M, Webster M *et al*: **The human colon cancer methylome shows similar hypo- and hypermethylation at conserved tissue-specific CpG island shores**. *Nature Genetics* 2009, **41**:178.
4. Ziller MJ, Gu H, Müller F, Donaghey J, Tsai LTY, Kohlbacher O, De Jager PL, Ro [illegible] ett DA, Bernstein BE *et al*: **Charting a dynamic DNA methylation landscape of th[illegible] genome**. *Nature* 2013, **500**:477.
5. Maunakea AK, Nagarajan RP, Bilenky M, Ballinger TJ, D'Souza C, Fouse SD, Johnson BE, Hong C, Nielsen C, Zhao Y *et al*: **Conserved role of intragenic DNA methylation in regulating alternative promoters**. *Nature* 2010, **466**:253.
6. Shenker N, Flanagan JM: **Intragenic DNA methylation: implications of this epigenetic mechanism for cancer research**. *British Journal Of Cancer* 2011, **106**:248.
7. Fogarty MJ: **Driven to decay: Excitability and synaptic abnormalities in amyotrophic lateral sclerosis**. *Brain Research Bulletin* 2018, **140**:318-333.
8. Kiernan MC, Vucic S, Cheah BC, Turner MR, Eisen A, Hardiman O, Burrell JR, Zoing MC: **Amyotrophic lateral sclerosis**. *The Lancet* 2011, **377**(9769):942-955.
9. Fogarty MJ: **Amyotrophic lateral sclerosis as a synaptopathy**. *Neural Regen Res* 2019, **14**(2):189-192.
10. Gratten J, Visscher PM: **Genetic pleiotropy in complex traits and diseases: implications for genomic medicine**. *Genome Medicine* 2016, **8**(1):78.
11. Watanabe K, Stringer S, Frei O, Umićević Mirkov M, de Leeuw C, Polderman TJC, van der Sluis S, Andreassen OA, Neale BM, Posthuma D: **A global overview of pleiotropy and genetic architecture in complex traits**. *Nature Genetics* 2019.
12. Li L, Xiong W-C, Mei L: **Neuromuscular Junction Formation, Aging, and Disorders**. *Annual Review of Physiology* 2018, **80**(1):159-188.
13. Zhao W, Beers DR, Hooten KG, Sieglaff DH, Zhang A, Kalyana-Sundaram S, Traini CM, Halsey WS, Hughes AM, Sathe GM *et al*: **Characterization of Gene Expression Phenotype in Amyotrophic Lateral Sclerosis Monocytes**. *JAMA neurology* 2017, **74**(6):677-685.
14. Steyn FJ, Lee K, Fogarty MJ, Veldhuis JD, McCombe PA, Bellingham MC, Ngo ST, Chen C: **Growth Hormone Secretion Is Correlated With Neuromuscular Innervation Rather Than Motor Neuron Number in Early-Symptomatic Male Amyotrophic Lateral Sclerosis Mice**. *Endocrinology* 2013, **154**(12):4695-4706.
15. Steyn FJ, Ngo ST, Lee JD, Leong JW, Buckley AJ, Veldhuis JD, McCombe PA, Chen C, Bellingham MC: **Impairments to the GH-IGF-I Axis in hSOD1G93A Mice Give Insight into Possible Mechanisms of GH Dysregulation in Patients with Amyotrophic Lateral Sclerosis**. *Endocrinology* 2012, **153**(8):3735-3746.
16. Dobrowolny G, Aucello M, Molinaro M, Musarò A: **Local expression of mIgf-1 modulates ubiquitin, caspase and CDK5 expression in skeletal muscle of an ALS mouse model**. *Neurological Research* 2008, **30**(2):131-136.
17. Dobrowolny G, Giacinti C, Pelosi L, Nicoletti C, Winn N, Barberi L, Molinaro M, Rosenthal N, Musarò A: **Muscle expression of a local Igf-1 isoform protects motor neurons in an ALS mouse model**. *The Journal of Cell Biology* 2005, **168**(2):193.
18. Palazzolo I, Stack C, Kong L, Musaro A, Adachi H, Katsuno M, Sobue G, Taylor JP, Sumner CJ, Fischbeck KH *et al*: **Overexpression of IGF-1 in muscle attenuates disease in a mouse model of spinal and bulbar muscular atrophy**. *Neuron* 2009, **63**(3):316-328.
19. Saccà F, Quarantelli M, Rinaldi C, Tucci T, Piro R, Perrotta G, Carotenuto B, Marsili A, Palma V, De Michele G *et al*: **A randomized controlled clinical trial of growth hormone in amyotrophic lateral sclerosis: clinical, neuroimaging, and hormonal results**. *Journal of Neurology* 2012, **259**(1):132-138.
20. Smith RA, Melmed S, Sherman B, France J, Munsat TL, Festoff BW: **Recombinant growth hormone treatment of amyotrophic lateral sclerosis**. *Muscle & Nerve* 1993, **16**(6):624-633.

21. Sorenson EJ, Windbank AJ, Mandrekar JN, Bamlet WR, Appel SH, Armon C, Barkhaus PE, Bosch P, Boylan K, David WS *et al*: **Subcutaneous IGF-1 is not beneficial in 2-year ALS trial**. *Neurology* 2008, **71**(22):1770.
22. Gerard M, Deleersnijder A, Demeulemeester J, Debyser Z, Baekelandt V: **Unraveling the Role of Peptidyl-Prolyl Isomerases in Neurodegeneration**. *Molecular Neurobiology* 2011, **44**(1):13-27.
23. Binder EB: **The role of FKBP5, a co-chaperone of the glucocorticoid receptor in the pathogenesis and therapy of affective and anxiety disorders**. *Psychoneuroendocrinology* 2009, **34**:S186-S195.
24. Blair LJ, Nordhues BA, Hill SE, Scaglione KM, O'Leary JC, III, Fontaine SN, Breydo L, Zhang B, Li P, Wang L *et al*: **Accelerated neurodegeneration through chaperone-mediated oligomerization of tau**. *The Journal of Clinical Investigation* 2013, **123**(10):4158-4169.
25. Maden M: **Retinoic acid in the development, regeneration and maintenance of the nervous system**. *Nat Rev Neurosci* 2007, **8**(10):755-765.
26. Boylan JF, Gudas LJ: **The level of CRABP-I expression influences the amounts and types of all- trans-retinoic acid metabolites in F9 teratocarcinoma stem cells**. *Journal of Biological Chemistry* 1992, **267**(30):21486-21491.
27. Delva L, Bastie J-N, Rochette-Egly C, Kraïba R, Balitrand N, Despouy G, Chambon P, Chomienne C: **Physical and Functional Interactions between Cellular Retinoic Acid Binding Protein II and the Retinoic Acid-Dependent Nuclear Complex**. *Molecular and Cellular Biology* 1999, **19**(10):7158.
28. Riancho J, Berciano MT, Ruiz-Soto M, Berciano J, Landreth G, Lafarga M: **Retinoids and motor neuron disease: Potential role in amyotrophic lateral sclerosis**. *Journal of the Neurological Sciences* 2016, **360**:115-120.
29. Andersson T, Sodersten E, Duckworth JK, Cascante A, Fritz N, Sacchetti P, Cervenka I, Bryja V, Hermanson O: **CXXC5 is a novel BMP4-regulated modulator of Wnt signaling in neural stem cells**. *J Biol Chem* 2009, **284**(6):3672-3681.
30. Kim MY, Kim HY, Hong J, Kim D, Lee H, Cheong E, Lee Y, Roth J, Kim DG, Min do S *et al*: **CXXC5 plays a role as a transcription activator for myelin genes on oligodendrocyte differentiation**. *Glia* 2016, **64**(3):350-362.
31. Ferraiuolo L, Meyer K, Sherwood TW, Vick J, Likhite S, Frakes A, Miranda CJ, Braun L, Heath PR, Pineda R *et al*: **Oligodendrocytes contribute to motor neuron death in ALS via SOD1-dependent mechanism**. *Proc Natl Acad Sci U S A* 2016, **113**(42):E6496-E6505.
